# Chromatograms: A Modular Infrastructure for Scalable Chromatographic Data Handling in R

**DOI:** 10.1021/acs.analchem.6c03926

**Published:** 2026-09-17

**Authors:** Philippine Louail, Laurent Gatto, Sebastian Gibb, Johannes Rainer

**Affiliations:** † Institute for Biomedicine, 198234 Eurac Research, Via A. Volta 21, 39100 Bolzano, Italy; ‡ Chair for Bioinformatics, Faculty of Mathematics and Computer Science, Friedrich Schiller University Jena, Ernst-Abbe-Platz 2, 07743 Jena, Germany; § Computational Biology and Bioinformatics, de Duve Institute, UCLouvain, Avenue Hippocrate 75, 1200 Brussels, Belgium; ∥ Department of Anaesthesiology and Intensive Care, University Medicine Greifswald, Ferdinand-Sauerbruch-Strasse 23A, 17475 Greifswald, Germany

## Abstract

Chromatographic traces are central to LC-MS analysis, yet most software treats them as transient intermediates inside a fixed pipeline rather than as data objects users can inspect and manipulate directly. In R, xcms provides the established infrastructure for large-scale LC-MS analysis, but the way it represents chromatograms limits what can be done with them once extracted. We present chromatograms, an open-source R/Bioconductor package and a core component of the R for mass spectrometry ecosystem, built on two design principles: interchangeable storage backends, which keep the analysis code independent of where the data reside (in memory, on disk, etc.), and delayed evaluation, which applies queued operations only to the chromatograms actually requested and processed at a time. Chromatograms was fully integrated into xcms hence enabling the efficient extraction of EICs for identified features, exemplified on public data, for which extraction of 4980 features across 4000 samples took only a few hours, against a full day with the legacy infrastructure. For targeted data analysis or inspection, a peak table from any external software, or published with a study, is enough to recover the corresponding chromatograms from the raw files, with annotation columns preserved; four published biomarkers were recovered from all 4063 samples of a cohort even without parallel processing in just under an hour. Chromatograms does not perform peak detection: it provides the infrastructure for working with chromatographic data once peaks are defined, giving a scalable foundation for targeted and untargeted LC-MS analysis in R.

## Graphical abstract

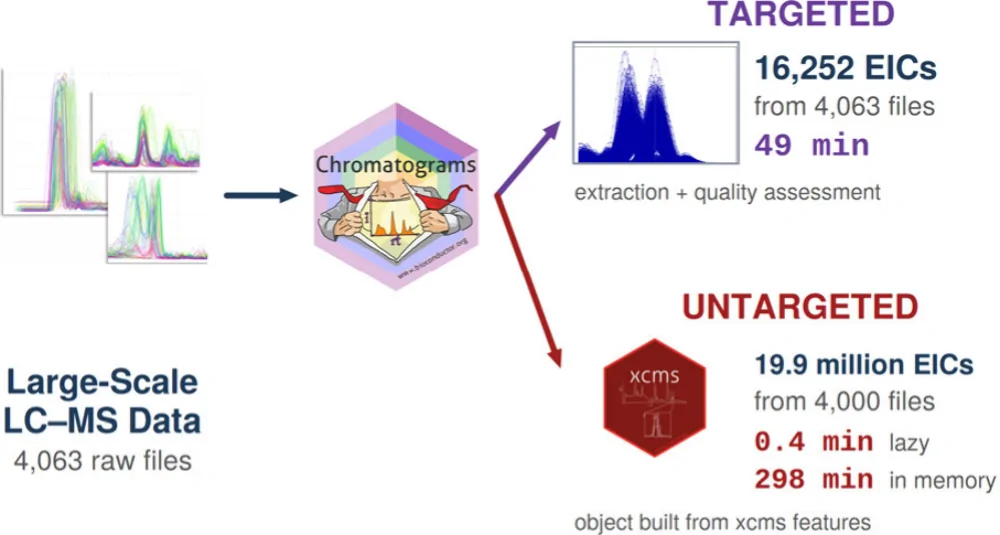

Chromatographic separation coupled to high-resolution mass spectrometry has become the central analytical technique for the untargeted analysis of complex mixtures, supporting metabolomics, exposomics, lipidomics, and small-molecule discovery. As cohort sizes, acquisition rates, and additional analytical dimensions such as ion mobility continue to inflate data volumes upward, the software infrastructure that handles chromatographic data must scale accordingly while remaining flexible enough to accommodate analyses that have not yet been imagined. Within R and Bioconductor, the xcms^1,2^ and MSnbase^3,4^ packages have historically provided the primary infrastructure, while the more recent Spectra^5^ package introduced a modular backend system and lazy evaluation for spectral data. Together, these two features substantially improve performance and memory efficiency for large-scale analyses, because operations are no longer forced to execute on the entire data set, and the underlying data can stay out of memory until they are actually needed.

Beyond R, the broader software landscape varies substantially in its treatment of the chromatographic data. Integrated platforms such as OpenMS^6,7^ , mzmine 4 ^8^, and MS-DIAL^9^ provide comprehensive processing pipelines, peak detection, alignment, and annotation, but treat chromatograms as intermediate products within these pipelines rather than as independently managed data objects. OpenMS stores chromatograms as a secondary collection within its spectrum-centric MSExperiment container; mzmine 4 ^8^ constructs extracted ion chromatograms transiently during the feature-building step; and MS-DIAL represents them within its own internal data model. Crucially, none of these platforms expose programmatic R or Python APIs that would allow users to manipulate chromatographic data interactively or to embed it in custom analysis scripts. They also offer no reusable building blocks for developers, who must therefore re-implement core chromatographic functionality each time they extend or adapt an existing pipeline. In R, the legacy MChromatograms class from xcms holds chromatographic data in memory and applies each operation to the whole object as soon as it is called, with no way to leave the data on disk and no support for parallel processing, limitations that become increasingly problematic as experiment sizes grow. In the Python ecosystem, memory-conscious strategies exist but operate on how data are read from disk rather than on how operations are applied to them: pyOpenMS^10^ holds data in an in-memory experiment container; tidyms^11^ and asari^12^ reduce RAM through on-demand mzML reads and disk-backed intermediates, respectively, but user-level operations such as filtering, imputation, or retention-time windowing execute, in all of these packages, immediately on the full in-memory data rather than being queued and applied only when the data are accessed. None also provide a single function that accepts a peak table produced by any other peak-picking software and uses it to extract chromatograms from the raw files for direct use in downstream quality assessment.

Two ideas from modern data engineering address these limitations directly. The first is lazy (delayed) evaluation: rather than running each operation immediately over the whole dataset, the software records what was requested and carries it out only when the data are needed, and only for the part of the data being used. The second is pluggable backends: the same analysis code runs unchanged whether the data sit in memory, are read from files on disk, or are derived on the fly from external sources. Both ideas are established in R/Bioconductor for spectral data through the Spectra package^5^ but have not yet been available for purely chromatographic data. Throughout the paper, we call the first mode “lazy” and the conventional immediate one “eager”.

Here we present Chromatograms, an R/Bioconductor package within the *R for Mass Spectrometry* (https://www.rformassspectrometry.org) ecosystem that applies and extends these design principles to chromatographic data. Peak detection is not part of Chromatograms; it remains the responsibility of established software such as xcms, mzmine, or MS-DIAL. Through *chromExtract­()*, combined with native integration with the MsQuality package^13^, it enables targeted extraction and automated quality assessment starting from a peak table produced by any upstream software. We quantify the scalability and lazy-evaluation advantages against the legacy R infrastructure on two public LC-HRMS datasets from MetaboLights: a ten-sample HILIC dataset (MTBLS8735) for controlled comparisons, and a 4063-sample reversed-phase cohort (MTBLS93) for cohort-scale performance and for a targeted reanalysis of the biomarkers the original study reported. By decoupling chromatographic data handling from any specific peak-picking pipeline, chromatograms let analysts combine tools freely across the LC-HRMS workflow and provides a scalable low-level foundation for interoperable chromatographic analysis in R.

## Experimental Section

### Package and Data Model

Chromatograms (version 1.3.3) is an R package distributed through Bioconductor. A chromatograms object wraps a chromData metadata table and per-chromatogram peak data (retention-time/intensity pairs) (Figure 1). The container represents any chromatographic trace (TIC, BPC, or EIC/XIC); all comparisons reported here use EICs.

**Figure 1:**
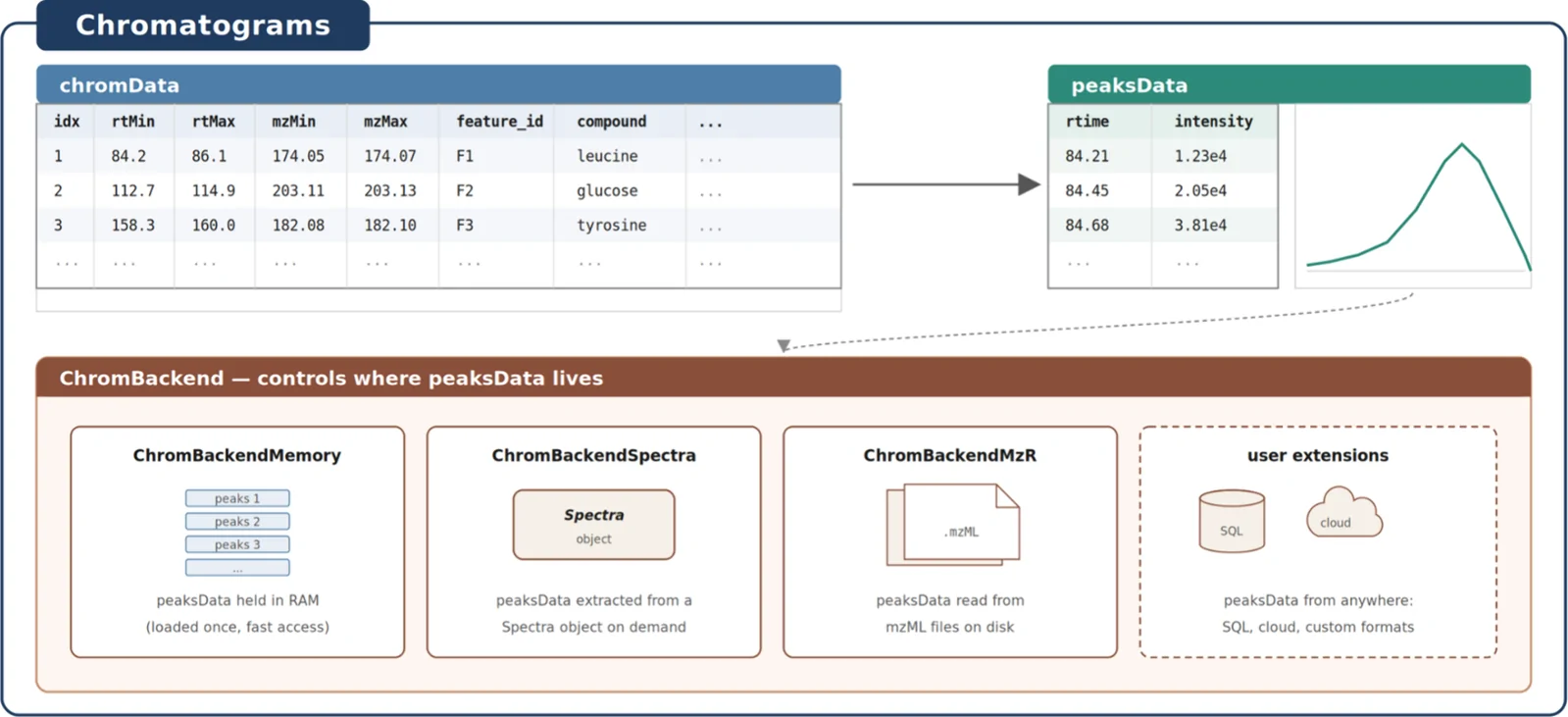
Anatomy of a Chromatograms object: a chromData table with one row per chromatogram, each row linked to a peaksData table of retention-time and intensity pairs, and the ChromBackend that determines where those values are read from, whether in memory, from disk, or from a *Spectra* object. Changing backend with *setBackend­()* leaves the tables and the analysis unchanged.

The data behind a Chromatograms object do not have to be held in memory. The aforementioned “pluggable backends” are what make this work in practice. The storage type is decided by the ChromBackend, and the same analysis code runs unchanged whichever one is in use. Three are currently provided in Chromatograms: ChromBackendMemory keeps the data in RAM, ChromBackendMzR reads them from mzML files on disk through mzR^14^, and ChromBackendSpectra derives them on demand from mass spectral data represented by a Spectra object^5^; users switch between them via *setBackend­()*. Chromatograms therefore covers both traces stored directly in mzML, as for SRM/MRM, and traces derived on demand from any source for which a Spectra backend exists: ion-mobility raw data, relational databases, and files streamed directly from public repositories, as used for the comparisons reported here^5^.

Chromatograms are now integrated as an additional chromatographic infrastructure in xcms: since version 4.11.2 its extraction functions *chromPeaksChromatograms­()*, *featureChromatograms­()*, and *chromatogram­()* can return Chromatograms objects.

Supported operations on a Chromatograms object include intensity-based filtering, missing-value interpolation, retention-time filtering, and peak-boundary detection, together with plotting functions. All data-manipulation operations are implemented in a “lazy” way through the processing queue described in the next subsection. Peak-quality assessment is provided externally by the MsQuality package^13^, which processes Chromatograms objects and computes standardized metrics (FWHM, signal-to-noise ratio, peak beta) without intermediate conversion.

### Lazy Evaluation and the Processing Queue

As introduced above, data-manipulation operations in Chromatograms are not executed immediately: they are queued inside the object and carried out only when data are accessed (the “lazy” concept). This confers two distinct advantages. First, transformations are applied only to the chromatograms the user actually requests; restricting the analysis to a region of interest therefore reduces both runtime and memory need. Second, operations can be applied to read-only sources (mzML files, *Spectra* objects) without writing a modified copy. For writable backends, queued transformations can be permanently applied (with the *applyProcessing­()* function).

### The chromExtract Functionality

The *chromExtract* function accepts a peak table imported from external sources such as plain text files or spreadsheets with columns specifying retention time and *m*/*z* boundaries together with one or more identifier columns that match chromatograms in the source object. It returns a new chromatograms object representing views on the original data, i.e., references to subsets of the MS data within the specified regions; any additional columns supplied in the input peak table (feature IDs, compound names, adduct annotations, external QC flags) are automatically carried through into the output chromData (Figure 1), preserving traceability from the original peak list to downstream analysis. Importantly, when combined with an on-disk backend (ChromBackendSpectra or ChromBackendMzR) and lazy evaluation, *chromExtract­()* operates without ever loading the complete raw dataset into memory: only the requested retention time/mz regions are read from disk when the resulting chromatograms are accessed. This makes the workflow possible on datasets that would not fit into RAM if loaded in full. Because the peak table can equally describe a handful of known compounds or every feature of an untargeted result, the same function supports the targeted and untargeted reanalysis of existing data.

### Data and Setup for the Performance Comparisons

The performance comparisons use two publicly available LC-HRMS datasets from the MetaboLights repository, chosen to probe different aspects of performance. MTBLS8735 is a ten-sample HILIC dataset in mzML format; its modest size allows controlled, repeated measurements in which the number of features, the number of files and the execution strategy are varied one at a time. MTBLS93 is a large reversed-phase LC-MS cohort of 4063 samples in netCDF file format from the TwinGene study^15^, used to test whether the behavior observed on the small dataset still holds at cohort scale. Because the two datasets differ in separation mode, instrument and file format, agreement between them also shows that the reported advantages are not specific to one type of data. All timings were recorded with serial (single-core) execution, so that the measured differences reflect the data structures themselves rather than the degree of parallelization. Workflow scripts (in Quarto format), input parameters, and complete R session information are provided as , making all reported results fully reproducible on any compatible system.

#### Comparison of Chromatographic Infrastructures within xcms (Supplementary Material 1)

xcms was selected as the reference implementation because it represents the state of the art for LC-MS chromatographic data processing and is widely used in the field, including beyond the R ecosystem. Within R, its MChromatograms class is the closest functional equivalent of a *Chromatograms* object, enabling a like-for-like comparison at the data-structure level. Comparison with non-R tools (OpenMS, mzmine, MS-DIAL) is not informative for this comparison, since they do not expose programmatic chromatogram objects to which the same analysis script could be applied. Three objects built from the same feature windows were compared, all produced by the same xcms extraction functions *featureChromatograms­()* and *chromatogram­()*: the legacy MChromatograms, a Chromatograms object as created (lazy, backed by ChromBackendSpectra), and the same object after its data have been loaded into memory with *setBackend­(ChromBackendMemory­())*. They were compared along three axes: the number of features extracted from a single file (10 to 5000, MTBLS8735), the number of files for a fixed set of features (MTBLS8735), and the full MTBLS93 cohort (4980 features, scaled up to 4000 files). Object-creation time and peak RAM footprint were recorded.

#### Lazy-Evaluation Comparison (Supplementary Material 2)

A representative three-step processing pipeline, intensity filtering, linear imputation of missing values, and retention-time windowing, was applied to the ten files of the MTBLS8735 dataset under six scenarios (Table 1) that combine the two backends (ChromBackendMemory, ChromBackendSpectra), two execution strategies (immediate: operation is executed immediately as it is called; lazy: operations are queued and applied only when data is accessed), and two access patterns (all chromatograms vs a single chromatogram). Immediate execution on the on-disk backend was not evaluated: it negates the memory advantage that motivates the use of the on-disk backend.

**Table 1:** Lazy-Evaluation Scenarios

scenario	backend	execution	access	what it isolates
(a)	ChromBackendMemory	immediate	all	baseline: immediate execution, all chromatograms
(b)	ChromBackendMemory	immediate	single	effect of access patterns under immediate execution
(c)	ChromBackendMemory	lazy	all	benefit of the processing queue alone
(d)	ChromBackendMemory	lazy	single	combined benefit of queue + single-chromatogram access
(e)	ChromBackendSpectra	lazy	all	on-disk worst case: all raw spectra read
(f)	ChromBackendSpectra	lazy	single	on-disk target workflow: minimal I/O + compute

#### Cohort-Scale Targeted Reanalysis (Supplementary Material 3)

The lipid biomarkers reported for the MTBLS93 cohort by the original study, *m*/*z* values, and retention times, were transcribed from that article into a plain peak table. The table was passed to *chromExtract­()*, which extracted the corresponding chromatograms directly from the raw files of all 4063 samples. The recovered peaks were quantified and quality-assessed with MsQuality functionalities, and the external annotation columns were carried out onto the chromData of the newly extracted EICs. Extraction time and memory use were recorded for an increasing number of files (10 to 4000).

## Results and Discussion

### Efficient Handling of Large Chromatographic Datasets

We first compare the Chromatogram representation with the legacy xcms representation on object-creation time and memory (Figure 2), then isolate the contribution of the processing queue across execution strategies and access patterns (Figure 3), and finally demonstrate the cohort-scale targeted workflow (Figure 4). The source code and report files with the full results are provided as Supplementary Materials 1, 2, and 3.

**Figure 2:**
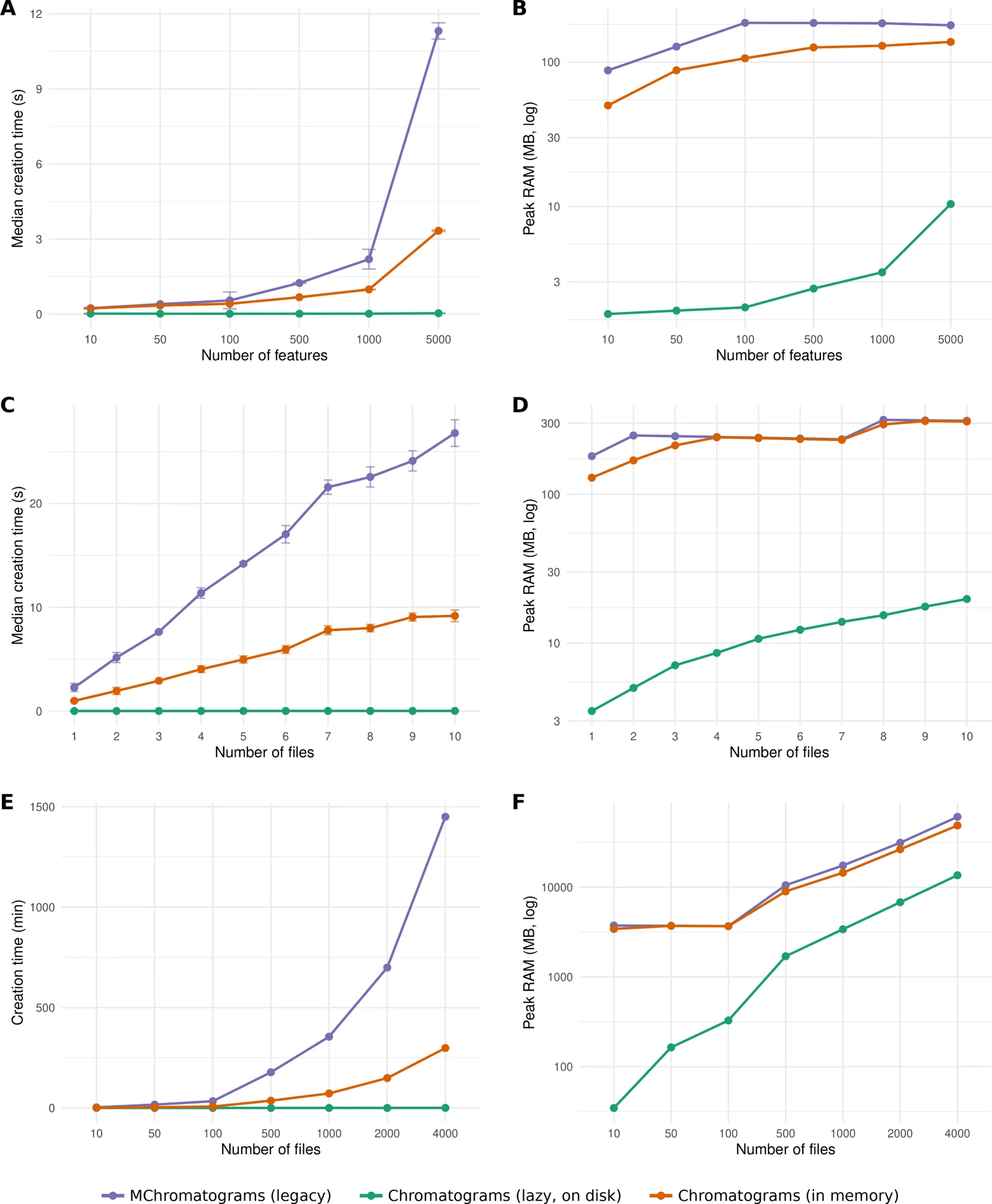
Object-creation time and memory usage for the legacy MChromatograms and the Chromatograms representation, from identical calls to *featureChromatograms­()* and *chromatogram­()*. Chromatograms is shown as created (lazy) and after loading into memory (eager). (A, B) Number of features, one file (MTBLS8735). (C, D) Number of files, 1000 features (MTBLS8735). (E, F) MTBLS93 cohort, 4980 features. Left: creation time; right: peak RAM (log scale).

**Figure 3:**
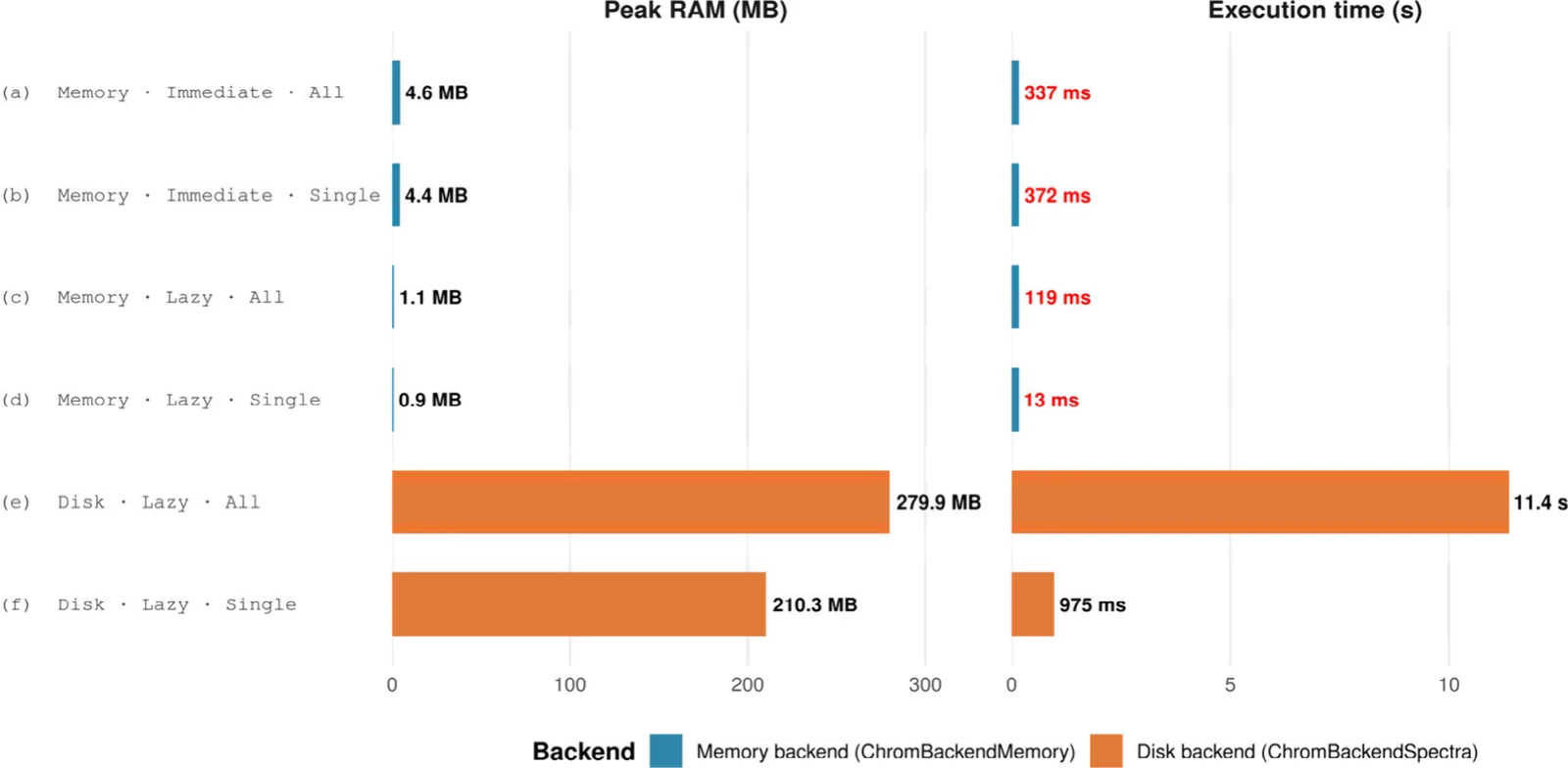
Execution time and peak memory for a three-step processing pipeline (intensity filtering, linear imputation, retention-time windowing) applied to MTBLS8735 under the six scenarios of Table 1. Peak RAM is shown on the left and execution time on the right; the labels (a) to (f) identify the scenarios, not panels. Times below 0.5 s are drawn at a minimum bar width and labeled in red.

**Figure 4:**
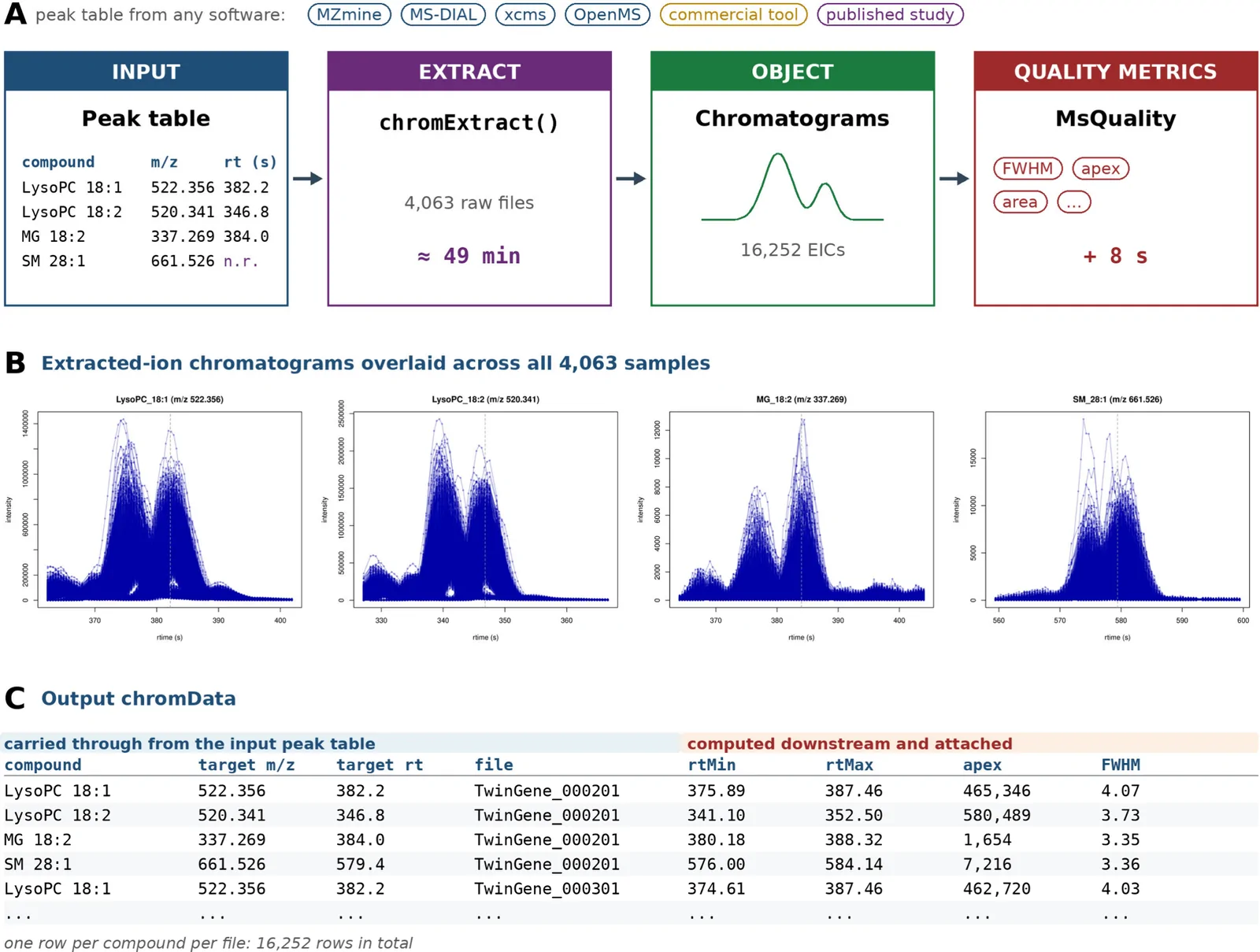
Cohort-scale targeted reanalysis with *chromExtract­()*, on the lipid biomarkers published for the MTBLS93 cohort. (A) The published peak table goes to *chromExtract­()*, giving 16252 chromatograms from 4063 samples, quality-assessed with MsQuality. SM 28:1 has no published retention time^15^ and was located from the data. (B) Chromatograms of each target overlaid across the cohort with *plotChromatogramsOverlay­()*; dashed line, annotated retention time. (C) Output chromData, one row per compound per file, with the input columns preserved and the boundaries and metrics attached.

#### Comparison of Chromatographic Infrastructures within xcms

Comparing the legacy MChromatograms with the new representation on identical calls to the same xcms extraction functions, *featureChromatograms­()* and *chromatogram­()*, the new infrastructure is cheaper in both time and memory requirements. A Chromatograms object is built in 0.02 to 0.04 s whether 10 or 5 000 features are requested, because at that point it holds only the chromatogram metadata and a reference to the raw data. Loading the data into memory with *setBackend­()* then pays the extraction cost, and it is this materialized object that gives the fair comparison against MChromatograms, which always holds its data in memory. On that basis, extracting the chromatograms of 5000 features from a single file takes 3.3 s and 138 MB, against 11.3 s and 180 MB for MChromatograms (Figure 2A,B), and the ordering is unchanged as the number of files grows (Figure 2C,D). At the cohort scale, the gap becomes decisive: extracting the 4980 feature chromatograms from 4000 files of the MTBLS93 twin study takes about 1450 min and 61 GB of peak RAM as MChromatograms, 298 min and 49 GB as the in-memory Chromatograms (Figure 2E,F; Supplementary Material 1). The advantage does not depend on the chromatography (HILIC or reversed phase) or on the file format (mzML or netCDF).

#### Lazy Evaluation Across Execution Strategies and Access Patterns

The six scenarios of Table 1 span the ways chromatographic data are handled in practice, from the immediate, whole-dataset execution used by most current software (scenarios a and b) to the queued, on-demand execution introduced here (scenarios c–f); their results are shown in Figure 3. The practical question for an analyst is what it costs to look at one (or a set of) compound(s) in a large data set. Immediate processing touches every chromatogram, whether or not it is needed. Simply queuing the three steps and running them in a single pass is already about three times faster at comparable memory (scenarios a versus c). The useful case is queuing them and then asking for one chromatogram: taking a subset carries the pending operations forward instead of evaluating them, so only that chromatogram is processed, about 26 times faster than the immediate baseline (scenarios a and b versus d), and the margin grows with the amount of data left untouched. Reading from disk follows the same logic: materializing a whole dataset is the most memory-hungry of the six scenarios (scenario e), whereas asking for one chromatogram reads one file and processes one trace (scenario e versus f). A dataset too large to fit in memory can therefore still be filtered, processed, and inspected one compound at a time, at interactive speed (Supplementary Material 2).

### Peak Extraction and Quality Assessment from External Peak Tables

We demonstrate the *chromExtract­()* function on a common use case: recovering the four coronary heart disease lipid biomarkers published for the MTBLS93 cohort directly from its raw data, starting from nothing but the m/z and retention time values printed in that article. The compound names and expected m/z and retention times were carried out automatically onto the resulting object, preserving traceability from the published table to the analysis that follows. One compound had no published retention time; it was located by extracting its trace over the whole elution window in ten files and taking the retention time of the median apex. The extracted chromatograms were quantified with *peakBoundary­()* and passed to the MsQuality package^13^ to obtain apex intensity, area under the chromatogram, and peak width at half-maximum, which were attached to the chromData alongside the imported annotations (Figure 4). Across the full cohort of 4063 samples, the extraction returned 16252 chromatograms in 49 min on a single core, of which the peak-boundary detection and the quality metrics together accounted for only 8 s, with peak memory staying in the single-digit gigabyte range and the resulting in-memory object occupying only 24 MB. The untargeted route to the same four compounds, rerunning peak detection, alignment, and correspondence on this cohort, instead takes many hours of processing and hundreds of gigabytes of intermediate storage (Supplementary Material 3). Overlaying the recovered traces across the cohort with *plotChromatogramsOverlay­()* (Figure 4B) makes sample-to-sample behavior visible directly: the annotated peak is present in every sample, and a subset of samples is displaced along the retention-time axis, a batch effect that the targeted extraction exposes without any further processing. The apex retention times of a single biomarker across the cohort fall into two well-separated clusters, a systematic deviation between subsets of the samples rather than random scattering (Supplementary Material 3).

Because *chromExtract­()* depends only on retention time and *m*/*z* ranges, a peak list from a commercial tool, mzmine^8^, MS-DIAL^9^, xcms, or a custom script all feed the same extraction and quality-assessment workflow, whatever software produced it. It also facilitates the reuse of published work: given the annotations a study reports and its deposited raw data, those compounds can be re-extracted and reassessed, in the original cohort or in new samples, without repeating the preprocessing that produced them.

## Conclusions

Treating chromatographic data as a first-class object changes what an analyst can do with a cohort. Work that previously required a compute cluster, or was simply not attempted, becomes possible on ordinary hardware: the chromatograms behind a published result can be inspected, requantified in new samples, or screened for batch effects without repeating the preprocessing that produced them. The gains reported here come from improved infrastructure and deferring computations rather than from faster algorithms, so they grow with the size of the dataset left untouched, and they hold whether the starting point is an untargeted feature list or a handful of known compounds. Peak picking is intentionally not part of Chromatograms; the package complements established peak-picking tools such as xcms rather than replacing them, and is intended to become the primary chromatographic data representation of xcms. The same functionality could also be built into targeted LC-MS tools such as TARDIS^16^, improving their performance considerably.

Beyond today’s use cases, Chromatograms is designed to be extended. The virtual ChromBackend class exposes a small, documented API that allows alternative data representations to be added without modifying the core package: a future backend wrapping a database, a cloud object store, or a column-oriented binary format can be loaded as an external package and used through the same Chromatograms API, as has already been done for Spectra, leaving the lazy queue and *chromExtract­()* unchanged. New functions to transform chromatographic data or to compute additional metrics can be integrated in the same way, while keeping all the functionality and advantages described here. As LC-HRMS data formats and analytical methods continue to evolve, this design lets Chromatograms accommodate new requirements through incremental contributions rather than disruptive re-implementations, ensuring the infrastructure scales with future analytical needs rather than being tied to today’s data formats and tools.

## Supplementary Material







## Data Availability

The Chromatograms package is available through Bioconductor at https://bioconductor.org/packages/Chromatograms. Development sources, issue tracker, and release tags are hosted at https://github.com/RforMassSpectrometry/Chromatograms. The package includes unit tests and user- and developer-oriented vignettes. All workflow scripts, input parameters, the published peak table used in the targeted workflow, and complete R session information are in the . The original Quarto source files utilized to generate the are hosted within the core software repository at: https://github.com/rformassspectrometry/Chromatograms/tree/main/inst/benchmarks. The LC–MS datasets analyzed in this study are available from the MetaboLights repository under the identifiers MTBLS8735 and MTBLS93. The package follows FAIR4RS^17,18^ principles for research software.

## Notes

Supplementary Material 1: demonstration of the xcms integration and performance comparison of the MChromatograms and Chromatograms representations (PDF)
Supplementary Material 2: lazy-evaluation comparison across execution strategies and access patterns (PDF)
Supplementary Material 3: interoperable, cohort-scale targeted reanalysis of a published dataset with *chromExtract­()*, *peakBoundary­()*, and MsQuality (PDF)
