# Supplementary material for: Chromatograms: A Modular Infrastructure for Scalable Chromatographic Data Handling in R
Source: Anal Chem. 2026 Sep 17;98(38):28337–44. doi: 10.1021/acs.analchem.6c03926 (PMC13629298; doi:10.1021/acs.analchem.6c03926)
Supplement: Supplementary file 1 [file ac6c03926_si_001.pdf]

Demonstration of *xcms* integration and performance comparisons

Supporting Information for: *Chromatograms: A modular infrastructure for scalable chromatographic data handling in R*

Philippine Louail[1,2,*], Laurent Gatto[3], Sebastian Gibb[4], Johannes Rainer[1]

[1] Institute for Biomedicine, Eurac Research, Bolzano, Italy [2] Chair for Bioinformatics, Friedrich-Schiller-University Jena, 07743 Jena, Germany [3] Computational Biology and Bioinformatics, de Duve Institute, UCLouvain, Brussels, Belgium [4] Department of Anaesthesiology and Intensive Care, University Medicine Greifswald, Greifswald, Germany

* Corresponding author: philippine.louail@outlook.com

Table of contents

Motivation S1
System information S2
Preprocessing S3
Part a - scaling the number of features (one file) S4
Part b - scaling the number of files (fixed features) S6
Part c - large-scale RPLC twin study S8
Key takeaways S12
Session info S12

Motivation

xcms version >= 4.11.2 PR #837 supports in addition to the legacy `MChromatograms` chromatographic data infrastructure defined in the *MSnbase* R package also the new functionality from the *Chromatograms* package, i.e., all functions to extract chromatographic data, such as `featureChromatograms()` or `chromatogram()` can return either a `Chromatograms` object (with the lazy `ChromBackendSpectra` backend) or the legacy `MChromatograms`. *lazy* data in this context means that no actual data is realized and loaded into memory at the time of object creation, while *eager* refers to the traditional approach where all data is processed and loaded at the time of object creation. The `featureChromatograms()` function extracts EICs for LC-MS features after a full LC-MS data preprocessing while `chromatogram()` allows to extract EICs for pre-defined m/z - retention time ranges.

On the **MTBLS8735** HILIC data set (preprocessed with *xcms*) we compare three objects built from the same features - each feature area padded by 10 s on each retention-time side and 20 ppm on each m/z side:

- **MChromatograms** - `return.type = "MChromatograms"`, legacy eager;
- **Chromatograms (lazy)** - `return.type = "Chromatograms"`, lazy `ChromBackendSpectra`;
- **Chromatograms (mem)** - initial lazy object creation with data being loaded into memory with `setBackend(ChromBackendMemory())`.

Part *a* scales the number of **features** in one file; part *b* scales the number of **files** for a fixed feature set.

To evaluate large-scale performance of `Chromatograms`, we use the **MTBLS93** RPLC LC-MS data set. The preprocessing of this data set with *xcms* is described in detail in the large-scale workflow of *Metabonaut*. The *xcms* `featureArea()` function was used to define the m/z - retention time windows for all 4,980 LC-MS features of the data set. Part *c* evaluates the processing time to extract these EICs from an increasing number of data files.

```
library(Chromatograms)
library(xcms)
library(Spectra)
library(MsBackendMetaboLights)
library(MsExperiment)
library(S4Vectors)
library(BiocParallel)
library(peakRAM)
library(ggplot2)

register(SerialParam())

methods <- c("MChromatograms", "Chromatograms (lazy)",
             "Chromatograms (mem)")
cols <- setNames(c("#7570B3", "#1B9E77", "#D95F02"), methods)
```

```
N_FEATURES <- c(10L, 50L, 100L, 500L, 1000L, 5000L)  # part a: features, one file
N_ITER     <- 5L                                      # timing iterations
CACHE_DIR  <- file.path("chrom_bm")
```

System information

```
knitr::kable(data.frame(
  Item = c("R version", "Platform", "Chromatograms", "xcms"),
  Value = c(R.version$version.string, R.version$platform,
            as.character(packageVersion("Chromatograms")),
            as.character(packageVersion("xcms")))),
  caption = "System information")
```

Table 1: System information

Item	Value
R version	R version 4.6.0 (2026-04-24)
Platform	x86_64-pc-linux-gnu
Chromatograms	1.3.3
xcms	4.11.2

Preprocessing

Every file is preprocessed to features once (`findChromPeaks()` + `groupChromPeaks()`, Metabonaut end-to-end settings) and cached. File subsets for the scaling use `filterFile(..., keepFeatures = TRUE)`.

```
dir.create(CACHE_DIR, showWarnings = FALSE, recursive = TRUE)
cache <- file.path(CACHE_DIR, "hilic_xe_all.rds")
xe_full <- if (file.exists(cache)) readRDS(cache) else {
  sps <- Spectra(source = MsBackendMetaboLights(), mtblsId = "MTBLS8735",
                 assayName = paste0("a_MTBLS8735_LC-MS_positive_",
                                    "hilic_metabolite_profiling.txt"),
                 filePattern = ".mzML")
  sps <- filterRt(sps, c(10, 240))
  f <- unique(dataOrigin(sps))
  mse <- MsExperiment()
  spectra(mse) <- sps
  sampleData(mse) <- DataFrame(sample_name = basename(f), dataOrigin = f,
                               phenotype = "sample")
  mse <- linkSampleData(mse, with = "sampleData.dataOrigin = spectra.dataOrigin")
  mse <- findChromPeaks(mse, CentWaveParam(peakwidth = c(1, 8), ppm = 15,
                                           integrate = 2), chunkSize = 2)
  mse <- groupChromPeaks(mse, PeakDensityParam(
           sampleGroups = sampleData(mse)$phenotype, minFraction = 0.5,
           binSize = 0.01, ppm = 10, bw = 1.8))
  saveRDS(mse, cache)
  mse
}
```

```
## median time, median peak RAM and object size of the three return types over
## N_ITER builds, for `sel` features of `xe` (areas padded 30 s / 20 ppm)
bench_cell <- function(xe, sel) {
  ex <- median(featureDefinitions(xe)$mzmed) * 20e-6
  one <- function(type, memory = FALSE) {
    t <- r <- numeric(N_ITER)
    for (i in seq_len(N_ITER)) {
      p <- peakRAM(o <- {
        x <- featureChromatograms(xe, features = sel, expandRt = 10,
                                  expandMz = ex, return.type = type)
        if (memory) setBackend(x, ChromBackendMemory()) else x
      })
      t[i] <- p$Elapsed_Time_sec
      r[i] <- p$Peak_RAM_Used_MiB
    }
    data.frame(median_s = median(t), sd_s = sd(t), peak_MB = median(r),
               size_MB = as.numeric(object.size(o)) / 1024^2)
  }
  data.frame(method = factor(methods, methods),
             rbind(one("MChromatograms"), one("Chromatograms"),
```

```r
                    one("Chromatograms", memory = TRUE)))
}

plot_metric <- function(d, x, y, ylab, log_y = FALSE) {
  p <- ggplot(d, aes(factor(.data[[x]]), .data[[y]], colour = method,
                     group = method)) +
    geom_line(linewidth = 1) + geom_point(size = 2.5) +
    scale_colour_manual(values = cols) +
    labs(x = if (x == "n_features") "Number of features" else "Number of files",
         y = ylab, colour = "Return type") +
    theme_minimal(base_size = 13)
  if (y == "median_s")
    p <- p + geom_errorbar(aes(ymin = pmax(median_s - sd_s, 0),
                               ymax = median_s + sd_s), width = 0.2, alpha = 0.6)
  if (log_y) p + scale_y_log10() else p
}
```

Part a - scaling the number of features (one file)

```r
xe1 <- filterFile(xe_full, 1L, keepFeatures = TRUE)
set.seed(1)
fids1 <- sample(rownames(featureDefinitions(xe1)))   # shuffled: random, nested
res_a <- do.call(
    rbind,
    lapply(N_FEATURES[N_FEATURES <= length(fids1)],
           function(k) cbind(bench_cell(xe1, fids1[seq_len(k)]), n_features = k)))
```

Subsets of 6582 features (in random order) extracted from one file.

```r
plot_metric(res_a, "n_features", "median_s", "Median creation time (s)")
```

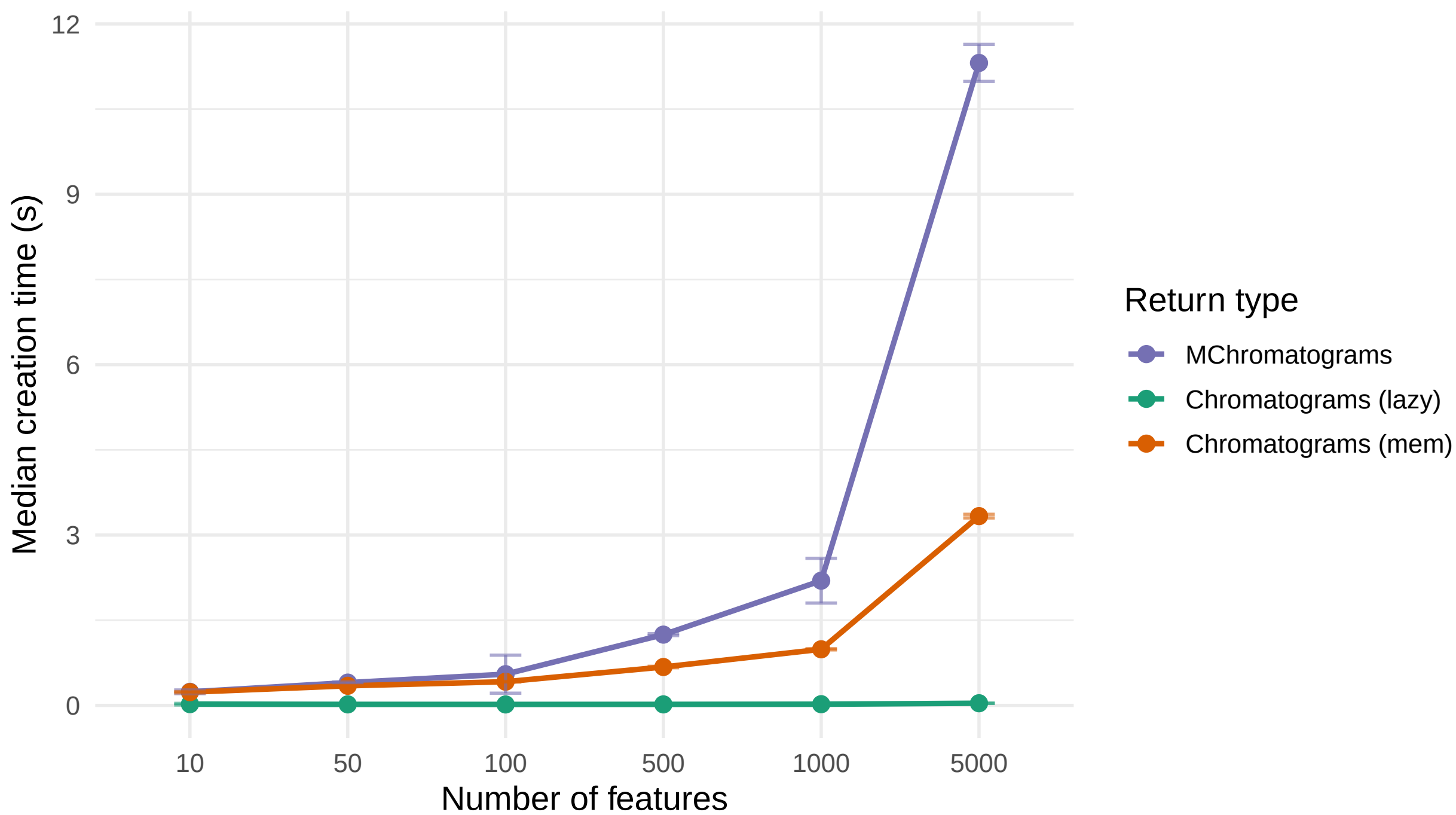

Figure 1: Median creation time vs number of features (one file).

```
plot_metric(res_a, "n_features", "peak_MB", "Peak RAM (MB, log)", log_y = TRUE)
```

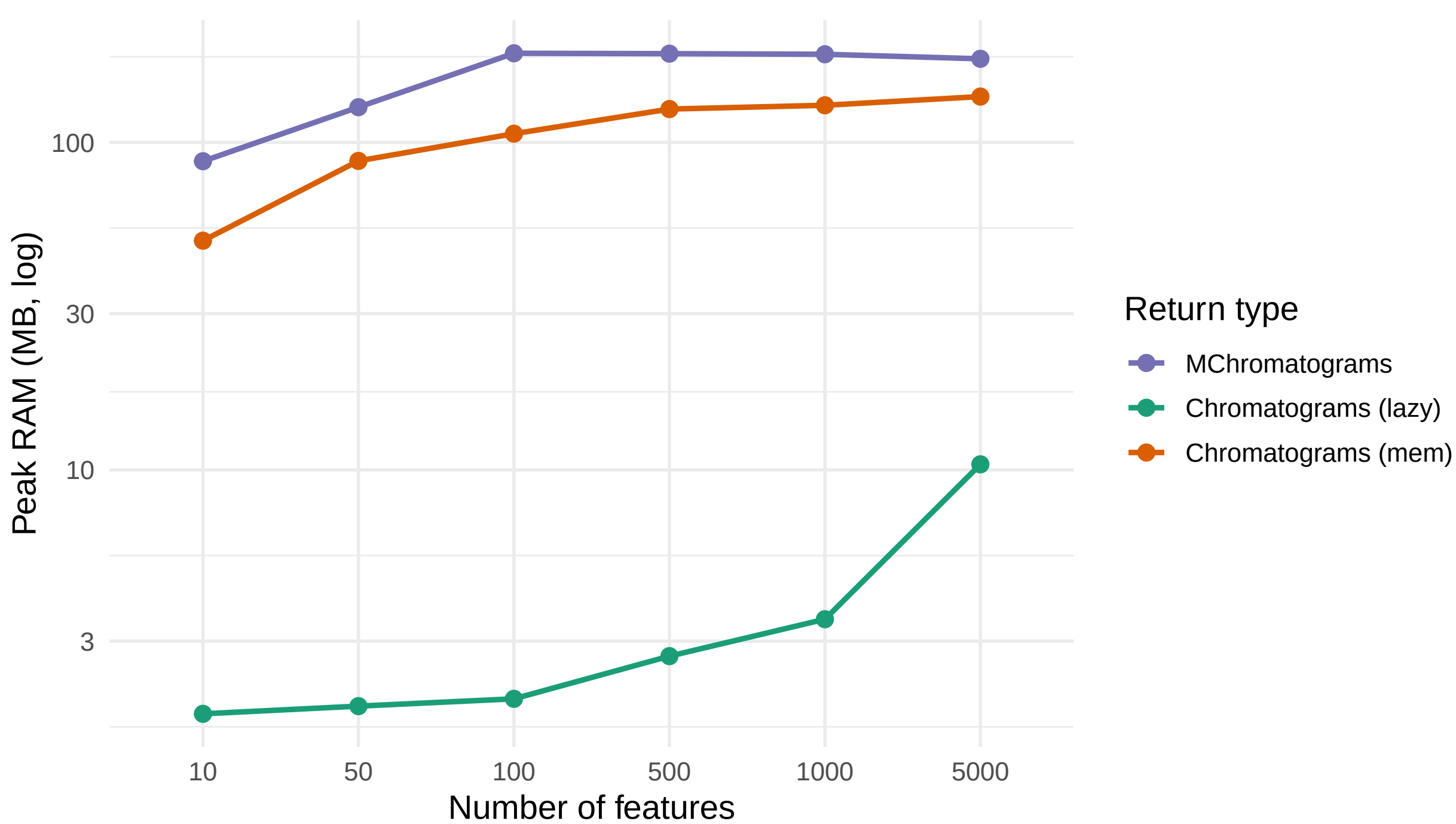

Figure 2: Peak RAM vs number of features (one file). Log scale.

Table 2

Features	Return type	Median time (s)	SD (s)	Peak RAM (MB)	Size (MB)
10	MChromatograms	0.239	0.033	87.6	0.06
10	Chromatograms (lazy)	0.022	0.014	1.8	0.19
10	Chromatograms (mem)	0.233	0.012	50.1	0.03
50	MChromatograms	0.401	0.011	128.1	0.25
50	Chromatograms (lazy)	0.018	0.001	1.9	0.25
50	Chromatograms (mem)	0.345	0.004	87.8	0.12
100	MChromatograms	0.550	0.335	187.1	0.50
100	Chromatograms (lazy)	0.017	0.001	2.0	0.32
100	Chromatograms (mem)	0.417	0.005	106.3	0.24
500	MChromatograms	1.246	0.017	186.6	2.45
500	Chromatograms (lazy)	0.018	0.000	2.7	0.75
500	Chromatograms (mem)	0.676	0.013	126.4	1.17
1000	MChromatograms	2.196	0.394	185.8	4.90
1000	Chromatograms (lazy)	0.021	0.003	3.5	1.27
1000	Chromatograms (mem)	0.988	0.010	129.8	2.35
5000	MChromatograms	11.313	0.326	180.0	24.41
5000	Chromatograms (lazy)	0.038	0.002	10.4	5.47
5000	Chromatograms (mem)	3.333	0.032	138.0	11.69

Part b - scaling the number of files (fixed features)

For a fixed number of features (n = 8329) evaluate performance extracting EICs from an increasing number of files.

```
set.seed(1)
res_b <- do.call(rbind, lapply(seq_along(fileNames(xe_full)), function(n) {
  xe <- filterFile(xe_full, seq_len(n), keepFeatures = TRUE)
  fids <- rownames(featureDefinitions(xe))
  cbind(bench_cell(xe, sample(fids, min(1000L, length(fids)))), n_files = n)
}))
```

```
plot_metric(res_b, "n_files", "median_s", "Median creation time (s)")
```

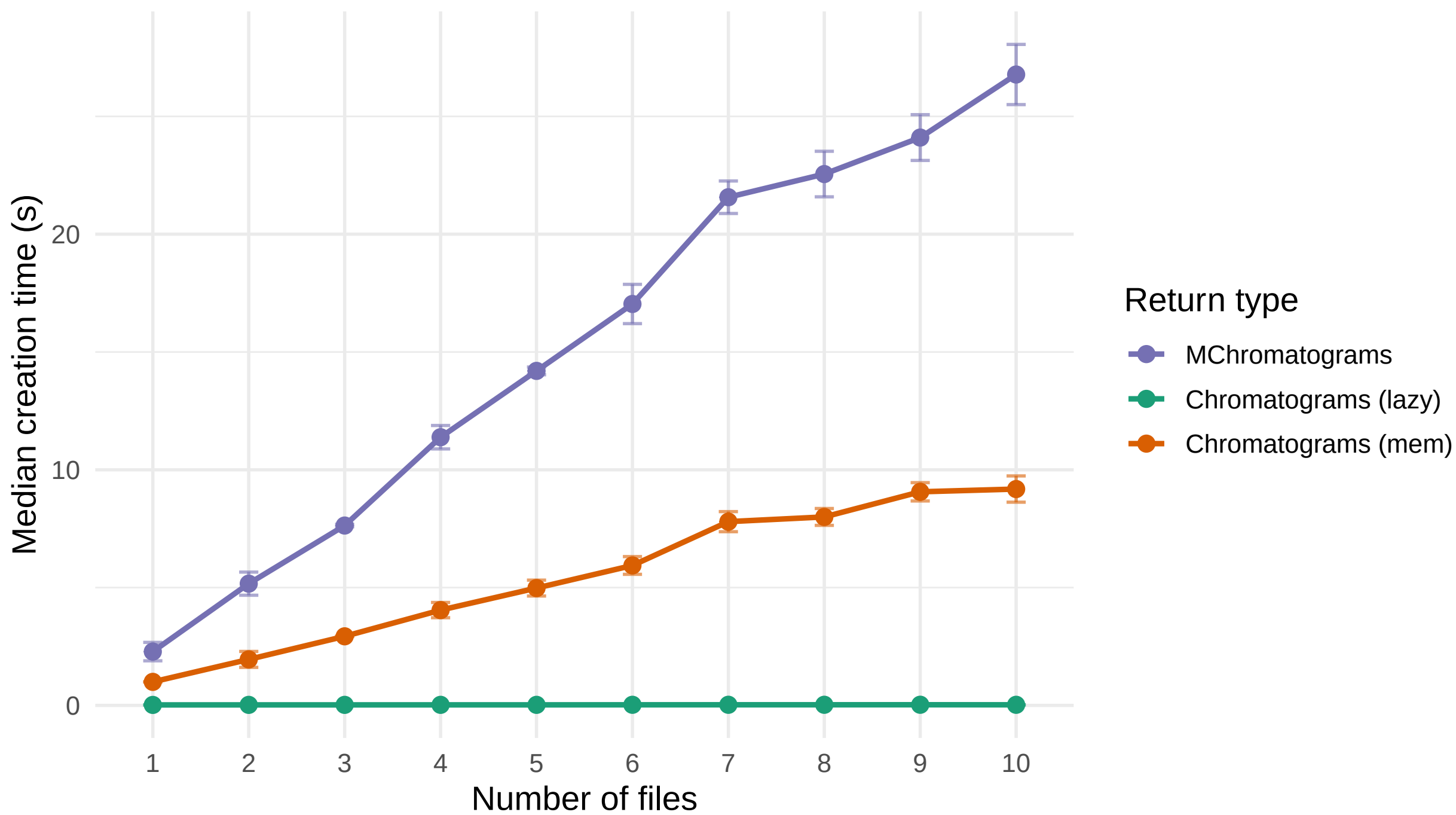

Figure 3: Median creation time vs number of files (1000 random features).

```
plot_metric(res_b, "n_files", "peak_MB", "Peak RAM (MB, log)", log_y = TRUE)
```

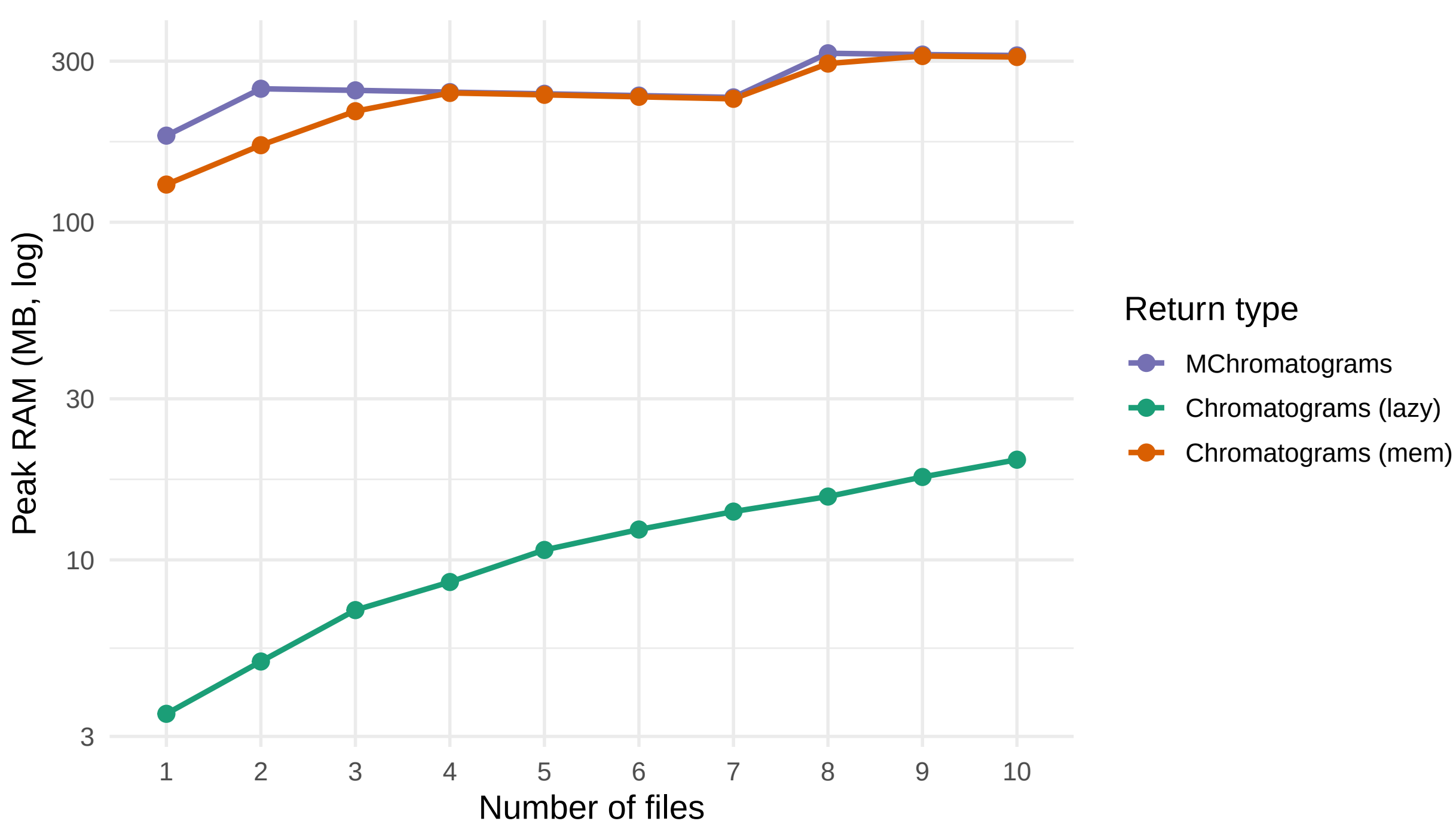

Figure 4: Peak RAM vs number of files (1000 random features). Log scale.

Table 3

Files	Return type	Median time (s)	SD (s)	Peak RAM (MB)	Size (MB)
1	MChromatograms	2.283	0.391	180.5	4.90
1	Chromatograms (lazy)	0.020	0.001	3.5	1.27
1	Chromatograms (mem)	0.998	0.008	129.3	2.35
2	MChromatograms	5.167	0.491	248.5	9.70
2	Chromatograms (lazy)	0.022	0.001	5.0	2.30
2	Chromatograms (mem)	1.952	0.338	169.1	4.58
3	MChromatograms	7.634	0.052	245.8	14.50
3	Chromatograms (lazy)	0.022	0.001	7.1	3.39
3	Chromatograms (mem)	2.932	0.010	213.2	6.88
4	MChromatograms	11.383	0.498	242.9	19.37
4	Chromatograms (lazy)	0.023	0.001	8.6	4.48
4	Chromatograms (mem)	4.045	0.323	241.5	9.25
5	MChromatograms	14.198	0.164	240.1	24.21
5	Chromatograms (lazy)	0.023	0.001	10.7	5.57
5	Chromatograms (mem)	4.980	0.338	238.4	11.57
6	MChromatograms	17.038	0.834	237.2	29.07
6	Chromatograms (lazy)	0.025	0.002	12.3	6.66
6	Chromatograms (mem)	5.940	0.376	235.2	13.93
7	MChromatograms	21.569	0.689	234.3	34.02
7	Chromatograms (lazy)	0.026	0.002	13.9	7.75
7	Chromatograms (mem)	7.799	0.426	232.0	16.37
8	MChromatograms	22.553	0.967	316.5	38.84
8	Chromatograms (lazy)	0.026	0.001	15.4	8.84
8	Chromatograms (mem)	7.999	0.358	295.0	18.68
9	MChromatograms	24.102	0.972	313.7	43.73
9	Chromatograms (lazy)	0.027	0.002	17.6	9.92
9	Chromatograms (mem)	9.066	0.389	310.6	21.07
10	MChromatograms	26.778	1.277	311.9	48.54
10	Chromatograms (lazy)	0.025	0.001	19.8	11.01
10	Chromatograms (mem)	9.182	0.557	308.6	23.37

Part c - large-scale RPLC twin study

Evaluate the performance extracting a fixed number of EICs (n = 4,980) from an increasing number of files of the large RPLC **MTBLS93** LC-MS data set. The *xcms* `chromatogram()` function with parameter `aggregationFun = "max"` and the pre-defined features' m/z - retention time windows is used to extract the EICs, using either the legacy `MChromatograms` or the new `Chromatograms` infrastructure.

```r
#' Loading the feature's m/z - retention time windows
fa <- read.table("twins-feature-area.txt", sep = "\t", header = TRUE)
```

Note: running the code in this section requires downloading and caching the full MS data (~ 4,000 CDF files) of the MTBLS93 data set from MetaboLights and requires approximately 600GB of disk

space.

```
library(MsBackendMetaboLights)
library(MsExperiment)
library(MsIO)
library(xcms)

#' Retrieve the MS1 data from the MetaboLights data set
mlp <- MetaboLightsParam(mtblsId = "MTBLS93", filePattern = "01.CDF$")

twins <- readMsObject(MsExperiment(), mlp, keepOntology = FALSE,
                      keepProtocol = FALSE, simplify = FALSE)
twins
```

```
Object of class MsExperiment
 Spectra: MS1 (12082605)
 Experiment data: 4063 sample(s)
 Sample data links:
  - spectra: 4063 sample(s) to 12082605 element(s).
```

Running the performance comparison for extraction of feature EICs from an increasing number of samples/files. Note that EICs for the same 4980 windows are extracted from every file, hence extracting in total from 49,800 up to 19,920,000 EICs from the smallest (n = 10) to largest data subset (n = 4,000).

```
nmbrs <- c(10, 50, 100, 500, 1000, 2000, 4000)

res_c <- lapply(nmbrs, function(n) {
    a <- twins[seq_len(n)]
    rt <- as.matrix(fa[, c("rtmin", "rtmax")])
    rt[, 1L] <- rt[, 1L] - 10
    rt[, 2L] <- rt[, 2L] + 10
    mz <- as.matrix(fa[, c("mzmin", "mzmax")])
    res <- peakRAM(
        chromatogram(a, rt = rt, mz = mz, return.type = "MChromatograms",
                     aggregationFun = "max"),
        chromatogram(a, rt = rt, mz = mz, return.type = "Chromatograms",
                     aggregationFun = "max"),
        {
            setBackend(chromatogram(
                a, rt = rt, mz = mz, return.type = "Chromatograms",
                aggregationFun = "max"),
                backend = ChromBackendMemory())
        }
    )
    res$method <- factor(c("MChromatograms", "Chromatograms (lazy)",
                           "Chromatograms (mem)"), methods)
    res$n_files <- n
    res
```

```
})
res_c <- do.call(rbind, res_c)
res_c$minutes <- res_c$Elapsed_Time_sec / 60
```

```
plot_metric(res_c, "n_files", "minutes", "Creation time (min)")
```

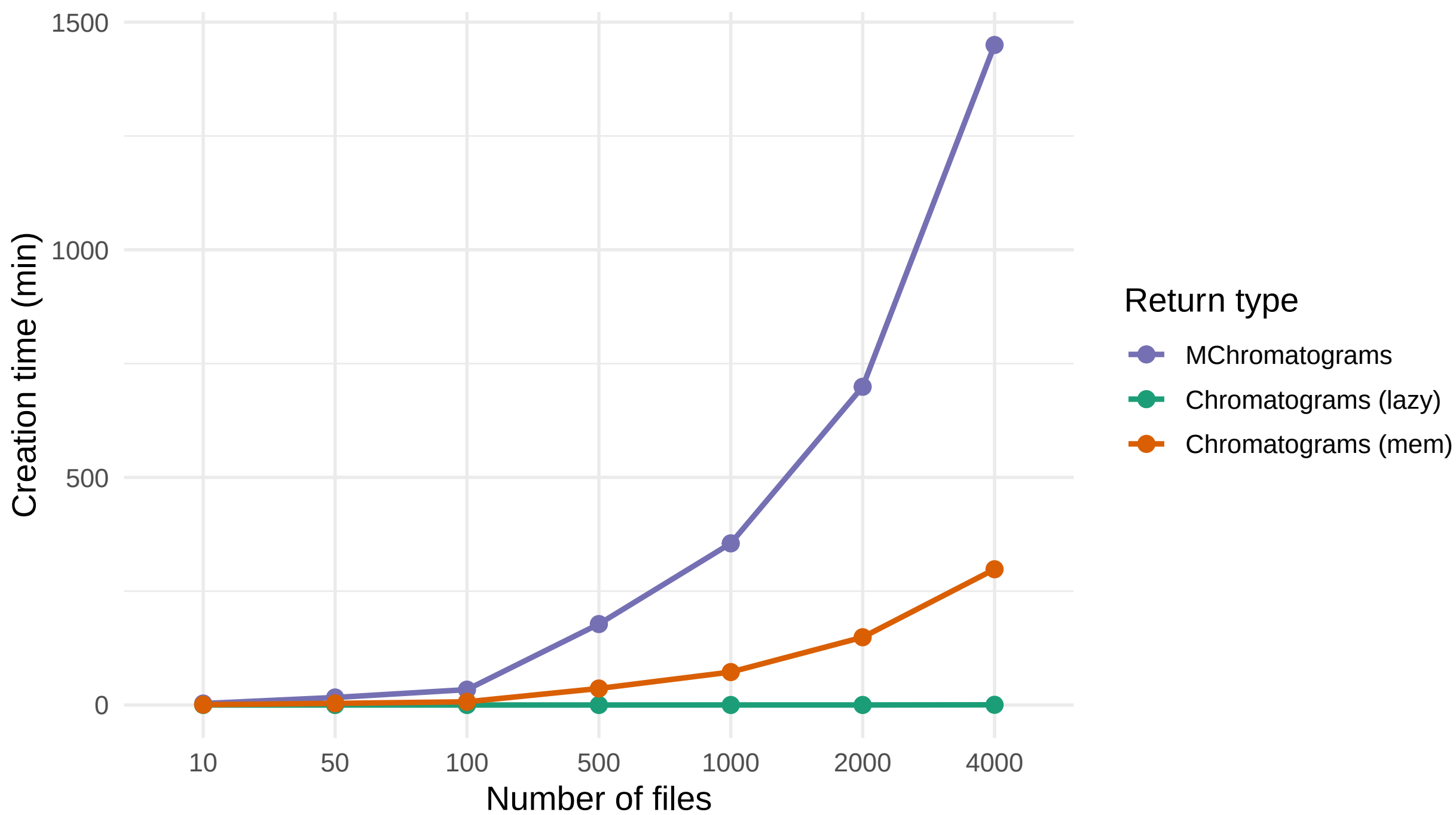

Figure 5: Median creation time vs number of files (4980 features).

```
plot_metric(res_c, "n_files", "Peak_RAM_Used_MiB", "Peak RAM (MB, log)",
            log_y = TRUE)
```

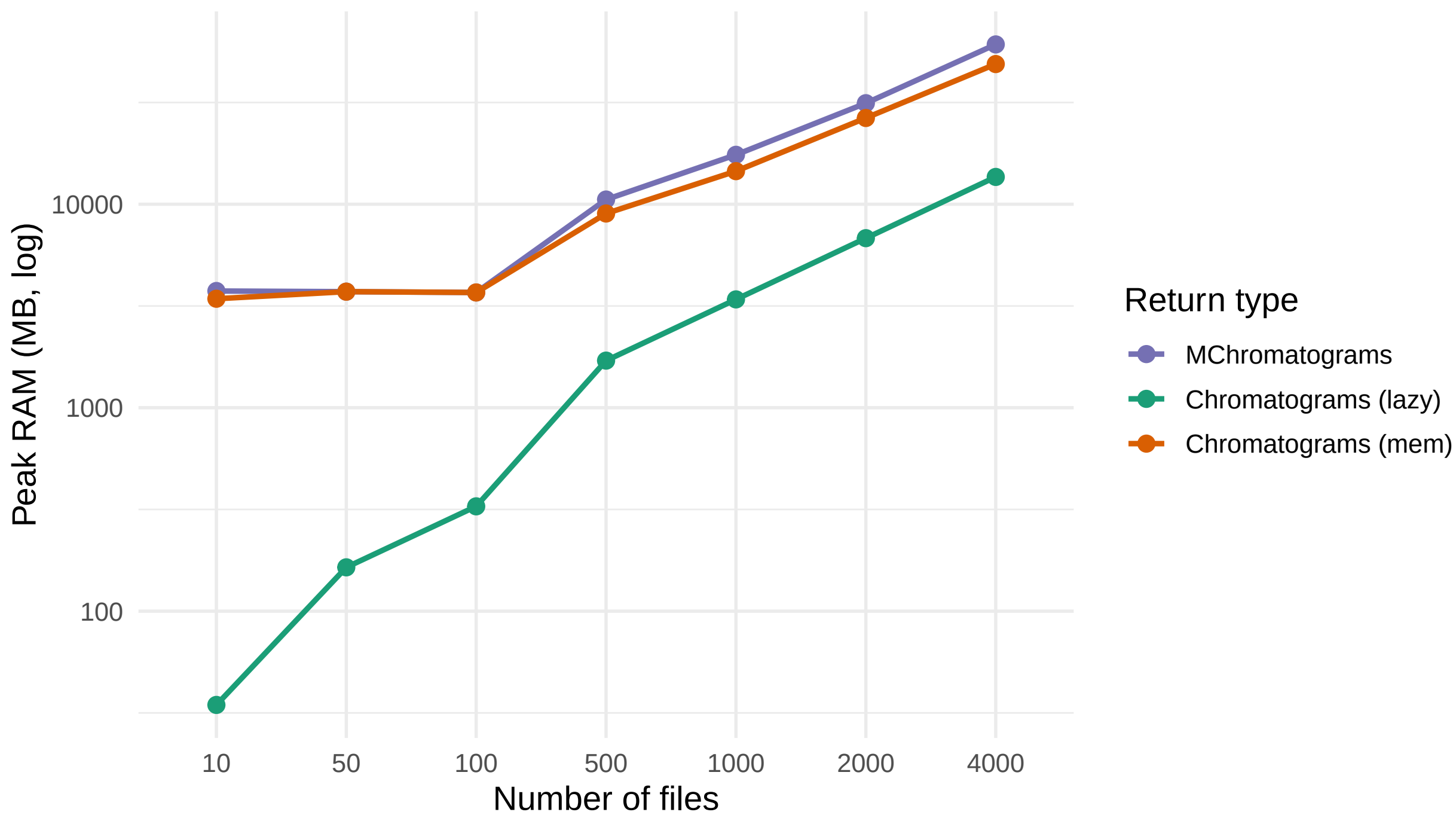

Figure 6: Peak RAM vs number of files (4980 features). Log scale.

Table 4

Files	Return type	Creation time (min)	Peak RAM (MB)	Size (MB)
10	MChromatograms	3.183	3746.2	81.9
10	Chromatograms (lazy)	0.001	34.6	8.8
10	Chromatograms (mem)	0.697	3432.1	85.5
50	MChromatograms	16.521	3718.5	408.2
50	Chromatograms (lazy)	0.001	164.2	44.2
50	Chromatograms (mem)	3.520	3718.5	427.7
100	MChromatograms	33.753	3684.0	816.0
100	Chromatograms (lazy)	0.002	327.5	88.5
100	Chromatograms (mem)	7.035	3684.0	855.5
500	MChromatograms	177.795	10547.4	4078.5
500	Chromatograms (lazy)	0.011	1703.6	442.5
500	Chromatograms (mem)	36.255	9004.6	4277.6
1000	MChromatograms	355.057	17498.7	8156.7
1000	Chromatograms (lazy)	0.022	3406.5	885.2
1000	Chromatograms (mem)	72.338	14542.5	8555.3
2000	MChromatograms	699.033	31381.6	16312.2
2000	Chromatograms (lazy)	0.044	6811.8	1770.3
2000	Chromatograms (mem)	148.835	26545.9	17109.7
4000	MChromatograms	1449.828	61033.5	32624.8
4000	Chromatograms (lazy)	0.416	13622.9	3540.6
4000	Chromatograms (mem)	298.191	48872.8	34220.1

Key takeaways

Returning a `Chromatograms` from `featureChromatograms()` (xcms #837) creates a lazy `ChromBackendSpectra` that is far less computationally demanding to build and holds far less peak RAM than the legacy `MChromatograms`, independent of the number of features or files. Materialising it with `setBackend(memory)` (i.e., loading the MS data into memory and aggregating the mass peak intensities to generate chromatographic data) pays the extraction, matching `MChromatograms`. The lazy advantage scales with the deferred extraction cost - modest here (narrow feature windows), large for wide windows or big feature sets.

Performance advantages of `Chromatograms` is independent on the type of LC-MS data set (HILIC or RPLC) or MS data file format (the first test data set used mzML files, the second, large-scale data set files in CDF format).

Session info

```r
sessionInfo()
```

```
R version 4.6.0 (2026-04-24)
Platform: x86_64-pc-linux-gnu
Running under: Arch Linux

Matrix products: default
BLAS:   /home/jo/R/local/R-4.6.0/lib64/R/lib/libRblas.so
LAPACK: /usr/lib/liblapack.so.3.12.0  LAPACK version 3.12.0

locale:
 [1] LC_CTYPE=en_US.UTF-8       LC_NUMERIC=C
 [3] LC_TIME=en_US.UTF-8        LC_COLLATE=en_US.UTF-8
 [5] LC_MONETARY=en_US.UTF-8    LC_MESSAGES=en_US.UTF-8
 [7] LC_PAPER=en_US.UTF-8       LC_NAME=C
 [9] LC_ADDRESS=C               LC_TELEPHONE=C
[11] LC_MEASUREMENT=en_US.UTF-8 LC_IDENTIFICATION=C

time zone: Europe/Rome
tzcode source: system (glibc)

attached base packages:
[1] stats4    stats     graphics  grDevices utils     datasets  methods
[8] base

other attached packages:
 [1] MsIO_0.0.17                 ggplot2_4.0.3
 [3] peakRAM_1.0.2               MsExperiment_1.14.0
 [5] MsBackendMetaboLights_1.6.1 Spectra_1.22.2
 [7] S4Vectors_0.50.1            BiocGenerics_0.58.1
 [9] generics_0.1.4              xcms_4.11.2
[11] Chromatograms_1.3.3         ProtGenerics_1.44.0
[13] BiocParallel_1.46.0
```

```
loaded via a namespace (and not attached):
  [1] DBI_1.3.0                     httr2_1.3.0
  [3] rlang_1.3.0                   magrittr_2.0.5
  [5] clue_0.3-68                   MassSpecWavelet_1.78.2
  [7] otel_0.2.0                    RSQLite_3.53.3
  [9] matrixStats_1.5.0             compiler_4.6.0
 [11] PTMods_1.0.0                  vctrs_0.7.3
 [13] reshape2_1.4.5                stringr_1.6.0
 [15] pkgconfig_2.0.3               MetaboCoreUtils_1.20.1
 [17] crayon_1.5.3                  fastmap_1.2.0
 [19] dbplyr_2.6.0                  XVector_0.52.0
 [21] labeling_0.4.3                rmarkdown_2.31
 [23] preprocessCore_1.74.0         bit_4.6.0
 [25] purrr_1.2.2                   xfun_0.60
 [27] MultiAssayExperiment_1.38.0 cachem_1.1.0
 [29] jsonlite_2.0.0                progress_1.2.3
 [31] blob_1.3.0                    rhdf5filters_1.24.0
 [33] DelayedArray_0.38.2           Rhdf5lib_2.0.0
 [35] parallel_4.6.0                prettyunits_1.2.0
 [37] cluster_2.1.8.3               R6_2.6.1
 [39] stringi_1.8.7                 RColorBrewer_1.1-3
 [41] limma_3.68.4                  GenomicRanges_1.64.0
 [43] Rcpp_1.1.2                    Seqinfo_1.2.0
 [45] SummarizedExperiment_1.42.0 iterators_1.0.14
 [47] knitr_1.51                    IRanges_2.46.0
 [49] Matrix_1.7-5                  igraph_2.3.3
 [51] tidyselect_1.2.1              dichromat_2.0-1
 [53] abind_1.4-8                   yaml_2.3.12
 [55] doParallel_1.0.17             codetools_0.2-20
 [57] affy_1.90.0                   curl_7.1.0
 [59] lattice_0.22-9                tibble_3.3.1
 [61] plyr_1.8.9                    withr_3.0.3
 [63] Biobase_2.72.0                S7_0.2.2
 [65] evaluate_1.0.5                BiocFileCache_3.2.0
 [67] alabaster.schemas_1.12.0      filelock_1.0.3
 [69] pillar_1.11.1                 affyio_1.82.0
 [71] BiocManager_1.30.27           MatrixGenerics_1.24.0
 [73] foreach_1.5.2                 MSnbase_2.37.0
 [75] MALDIquant_1.22.3             ncdf4_1.24
 [77] hms_1.1.4                     alabaster.base_1.12.1
 [79] scales_1.4.0                  glue_1.8.1
 [81] MsFeatures_1.20.0             lazyeval_0.2.3
 [83] tools_4.6.0                   mzID_1.50.0
 [85] data.table_1.18.4             QFeatures_1.22.0
 [87] vsn_3.80.0                    mzR_2.46.0
 [89] fs_2.1.0                      XML_3.99-0.23
 [91] rhdf5_2.56.0                  grid_4.6.0
```

```
 [93] impute_1.86.0              tidyr_1.3.2
 [95] MsCoreUtils_1.24.0         PSMatch_1.16.0
 [97] cli_3.6.6                  S4Arrays_1.12.0
 [99] dplyr_1.2.1                AnnotationFilter_1.36.0
[101] pcaMethods_2.4.0           gtable_0.3.6
[103] digest_0.6.39              SparseArray_1.12.2
[105] farver_2.1.2               memoise_2.0.1
[107] htmltools_0.5.9            lifecycle_1.0.5
[109] statmod_1.5.2              bit64_4.8.2
[111] MASS_7.3-66
```
