# Supplementary material for: Chromatograms: A Modular Infrastructure for Scalable Chromatographic Data Handling in R
Source: Anal Chem. 2026 Sep 17;98(38):28337–44. doi: 10.1021/acs.analchem.6c03926 (PMC13629298; doi:10.1021/acs.analchem.6c03926)
Supplement: Supplementary file 2 [file ac6c03926_si_002.pdf]

Effect of execution strategy, backend, and access pattern on a three-step chromatographic processing pipeline

Supporting Information for: *Chromatograms: A modular infrastructure for scalable chromatographic data handling in R*

Philippine Louail[1,2,*], Laurent Gatto[3], Sebastian Gibb[4], Johannes Rainer[1]

[1] Institute for Biomedicine, Eurac Research, Bolzano, Italy [2] Chair for Bioinformatics, Friedrich-Schiller-University Jena, 07743 Jena, Germany [3] Computational Biology and Bioinformatics, de Duve Institute, UCLouvain, Brussels, Belgium [4] Department of Anaesthesiology and Intensive Care, University Medicine Greifswald, Greifswald, Germany

* Corresponding author: philippine.louail@outlook.com

Table of contents

Motivation . . . S1
System Information . . . S2
1. Load and prepare data . . . S3
2. Processing pipeline . . . S4
3. Benchmark . . . S4
Scenario (a): in-memory + immediate + full access . . . S5
Scenario (b): in-memory + immediate + subset access . . . S5
Scenario (c): in-memory + lazy + full access . . . S5
Scenario (d): in-memory + lazy + subset access . . . S6
Scenario (e): on-disk + lazy + full access . . . S6
Scenario (f): on-disk + lazy + subset access . . . S7
4. Results . . . S7
Conclusion . . . S10
Immediate vs. lazy evaluation . . . S10
The cost of on-disk full access . . . S10
Practical workflow recommendations . . . S11
References . . . S11
Key results for manuscript . . . S11
Session Info . . . S12

Motivation

Legacy R/Bioconductor infrastructures for mass spectrometry data - most notably the *MChromatograms* class in *MSnbase*/*xcms* - apply every data-manipulation operation immediately and to the entire object. The *Chromatograms* package follows a different model, taking direct inspiration from the *Spectra*/*MSnbase* design[1][2]: a **lazy processing queue** defers computation, recording

[1]Rainer J, Gatto L, Gibb S. *Metabolites* 2022;12(2):173.
[2]Gatto L, Gibb S, Rainer J. *J Proteome Res.* 2020;19(4):1063–1069.

transformations rather than executing them, and applying them only when (and where) the data are finally materialised. When the user eventually accesses only a *subset* of the chromatograms, the queued operations are applied to that subset alone, never to the rest of the data.

To quantify the gain, we benchmark a single three-step processing pipeline under six scenarios that vary two dimensions:

- **Access pattern**: retrieve data from *all* chromatograms vs. a *single* chromatogram.
- **Execution strategy**: immediate (*applyProcessing()* after each step), lazy on in-memory data (*ChromBackendMemory*), or lazy on on-disk data (*ChromBackendSpectra*).

Scenario	Backend	Execution	Final access
(a)	*ChromBackendMemory*	Immediate	All chromatograms
(b)	*ChromBackendMemory*	Immediate	Single chromatogram
(c)	*ChromBackendMemory*	Lazy	All chromatograms
(d)	*ChromBackendMemory*	Lazy	Single chromatogram
(e)	*ChromBackendSpectra*	Lazy	All chromatograms
(f)	*ChromBackendSpectra*	Lazy	Single chromatogram

```
set.seed(123)
library(Chromatograms)
library(Spectra)
library(MsBackendMetaboLights)
library(peakRAM)
library(dplyr)
library(tidyr)
library(ggplot2)
```

System Information

```
sys_info <- data.frame(
  Property = c("CPU Cores", "R Version", "Platform"),
  Value = c(as.character(parallel::detectCores()),
            R.version$version.string, R.version$platform)
)
if (requireNamespace("Chromatograms", quietly = TRUE))
  sys_info <- rbind(sys_info, data.frame(
    Property = "Chromatograms version",
    Value = as.character(packageVersion("Chromatograms"))))
knitr::kable(sys_info, col.names = c("Property", "Value"),
             caption = "System information")
```

Table 1: System information

Property	Value
CPU Cores	24

Property	Value
R Version	R version 4.6.0 (2026-04-24)
Platform	x86_64-pc-linux-gnu
Chromatograms version	1.3.3

1. Load and prepare data

We load all available mzML files from MetaboLights (MTBLS8735) and prepare two starting objects:

- `chr_spectra` - backed by *ChromBackendSpectra* (on-disk, lazy). The raw m/z × intensity arrays remain on disk; data are only read when explicitly accessed.
- `chr_memory` - backed by *ChromBackendMemory* (in-memory), created via *setBackend()*, which materialises all chromatographic data into memory. This one-time cost is not included in the benchmarks below.

```
# Load public data from MetaboLights (MTBLS8735)
sps <- Spectra(
  source = MsBackendMetaboLights(),
  mtblsId = "MTBLS8735",
  assayName = paste0("a_MTBLS8735_LC-MS_positive_",
                     "hilic_metabolite_profiling.txt"),
  filePattern = ".mzML"
)

mzml_files <- unique(dataOrigin(sps))

# On-disk Chromatograms (ChromBackendSpectra)
chr_spectra <- Chromatograms(sps)

# In-memory Chromatograms (ChromBackendMemory)
# setBackend() reads and summarizes all spectra - a one-time cost.
chr_memory <- setBackend(chr_spectra, ChromBackendMemory())

knitr::kable(
  data.frame(
    Property = c("Files", "Total spectra", "Chromatograms",
                 "Size (on-disk)", "Size (in-memory)"),
    Value = c(length(mzml_files), length(sps), length(chr_spectra),
              format(object.size(chr_spectra), units = "Mb"),
              format(object.size(chr_memory), units = "Mb"))
  ),
  caption = "Loaded data summary"
)
```

Table 2: Loaded data summary

Property	Value
Files	10
Total spectra	17210
Chromatograms	10
Size (on-disk)	3.4 Mb
Size (in-memory)	0.3 Mb

2. Processing pipeline

All six scenarios apply the same three-step pipeline:

1. *filterPeaksData()* - remove data points with intensity <= 100.
2. *imputePeaksData()* - fill gaps by linear interpolation.
3. *filterPeaksData()* - restrict to a 200–400 s retention-time window.

The scenarios differ along two dimensions:

Execution strategy. In *immediate* mode, *applyProcessing()* is called after each step, forcing immediate evaluation across **all** chromatograms - this mirrors the behaviour of most existing software, where every operation reads and writes the full dataset. In *lazy* mode the transformations are queued, and the queue is evaluated only when actual data are requested - and only for the chromatograms requested.

Final data access. *Pipeline 1* calls *intensity(chr)*, which retrieves intensities from every chromatogram and therefore forces the queue (if any) to evaluate across the whole dataset. *Pipeline 2* calls *peaksData(chr[3])*, retrieving data from a single chromatogram. The `[` operator carries the pending queue forward without evaluating it, so *peaksData()* triggers evaluation only for the one chromatogram requested.

3. Benchmark

```
# Shared parameters for all scenarios
int_range <- c(100, Inf)
rt_window <- c(200, 400)

# Helper to extract elapsed time (s) and peak RAM (MB) from a peakRAM()
# result. peakRAM measures the high-water mark of resident RAM during the
# expression - more meaningful than bench::mark()'s cumulative profmem
# allocation for "how much memory does this need at any moment".
summarise_pr <- function(pr) {
  c(elapsed_s = as.numeric(pr$Elapsed_Time_sec),
    peak_mem_Mb = as.numeric(pr$Peak_RAM_Used_MiB))
}
```

Scenario (a): in-memory + immediate + full access

Baseline. *applyProcessing()* is called after each step, forcing immediate evaluation across all chromatograms - three full passes over the data. The final *intensity()* call simply reads already-processed data. This mirrors the behaviour of most traditional tools.

```
pr_a <- peakRAM({
  chr <- filterPeaksData(chr_memory, variables = "intensity",
                         ranges = int_range)
  chr <- applyProcessing(chr)
  chr <- imputePeaksData(chr, method = "linear")
  chr <- applyProcessing(chr)
  chr <- filterPeaksData(chr, variables = "rtime", ranges = rt_window)
  chr <- applyProcessing(chr)
  res <- intensity(chr)
})
res_a <- summarise_pr(pr_a)
res_a
```

```
  elapsed_s peak_mem_Mb
      0.337       4.600
```

Scenario (b): in-memory + immediate + subset access

Same as (a), but the final call requests data from a single chromatogram. The timing is comparable to (a) because the three *applyProcessing()* calls have already processed the full dataset - the subset at the end saves negligible time.

```
pr_b <- peakRAM({
  chr <- filterPeaksData(chr_memory, variables = "intensity",
                         ranges = int_range)
  chr <- applyProcessing(chr)
  chr <- imputePeaksData(chr, method = "linear")
  chr <- applyProcessing(chr)
  chr <- filterPeaksData(chr, variables = "rtime", ranges = rt_window)
  chr <- applyProcessing(chr)
  res <- peaksData(chr[3])
})
res_b <- summarise_pr(pr_b)
res_b
```

```
  elapsed_s peak_mem_Mb
      0.372       4.400
```

Scenario (c): in-memory + lazy + full access

No *applyProcessing()* calls - the three steps are queued. *intensity(chr)* then triggers evaluation of the full queue on **all** chromatograms at once. The total work is comparable to (a), but performed in a single pass rather than three.

```r
pr_c <- peakRAM({
  chr <- filterPeaksData(chr_memory, variables = "intensity",
                         ranges = int_range)
  chr <- imputePeaksData(chr, method = "linear")
  chr <- filterPeaksData(chr, variables = "rtime", ranges = rt_window)
  res <- intensity(chr)
})
res_c <- summarise_pr(pr_c)
res_c
```

```
  elapsed_s peak_mem_Mb
      0.119       1.100
```

Scenario (d): in-memory + lazy + subset access

The key scenario. The same three steps are queued, but *peaksData(chr[3])* requests data from a **single** chromatogram. The `[` operator subsets the object *without* evaluating the queue - it simply carries the pending operations forward. *peaksData()* then evaluates the queue **only** for that one chromatogram, skipping all others entirely.

```r
pr_d <- peakRAM({
  chr <- filterPeaksData(chr_memory, variables = "intensity",
                         ranges = int_range)
  chr <- imputePeaksData(chr, method = "linear")
  chr <- filterPeaksData(chr, variables = "rtime", ranges = rt_window)
  res <- peaksData(chr[3])
})
res_d <- summarise_pr(pr_d)
res_d
```

```
  elapsed_s peak_mem_Mb
      0.013       0.900
```

Scenario (e): on-disk + lazy + full access

Same lazy pipeline, but starting from *ChromBackendSpectra*. The data live on disk and are read only when *intensity()* triggers queue evaluation. The total cost includes disk I/O for all files plus processing of all chromatograms.

```r
pr_e <- peakRAM({
  chr <- filterPeaksData(chr_spectra, variables = "intensity",
                         ranges = int_range)
  chr <- imputePeaksData(chr, method = "linear")
  chr <- filterPeaksData(chr, variables = "rtime", ranges = rt_window)
  res <- intensity(chr)
})
res_e <- summarise_pr(pr_e)
res_e
```

```
  elapsed_s peak_mem_Mb
```

```
      11.377     279.900
```

Scenario (f): on-disk + lazy + subset access

The optimal scenario. The lazy queue is built on *ChromBackendSpectra*, and *peaksData(chr[3])* triggers evaluation for one chromatogram only. Only one file's raw spectra are read from disk, and only one chromatogram is processed through the full pipeline.

```r
pr_f <- peakRAM({
  chr <- filterPeaksData(chr_spectra, variables = "intensity",
                         ranges = int_range)
  chr <- imputePeaksData(chr, method = "linear")
  chr <- filterPeaksData(chr, variables = "rtime", ranges = rt_window)
  res <- peaksData(chr[3])
})
res_f <- summarise_pr(pr_f)
res_f
```

```
  elapsed_s peak_mem_Mb
      0.975     210.300
```

4. Results

```r
results <- data.frame(
  Scenario = c("(a)", "(b)", "(c)", "(d)", "(e)", "(f)"),
  Backend = c(rep("ChromBackendMemory", 4),
              rep("ChromBackendSpectra", 2)),
  Processing = c("Immediate", "Immediate", "Lazy", "Lazy", "Lazy", "Lazy"),
  Access = c("All", "Single", "All", "Single", "All", "Single"),
  Time_s = round(c(res_a["elapsed_s"], res_b["elapsed_s"],
                   res_c["elapsed_s"], res_d["elapsed_s"],
                   res_e["elapsed_s"], res_f["elapsed_s"]), 3),
  Peak_Mem_Mb = round(c(res_a["peak_mem_Mb"], res_b["peak_mem_Mb"],
                        res_c["peak_mem_Mb"], res_d["peak_mem_Mb"],
                        res_e["peak_mem_Mb"], res_f["peak_mem_Mb"]), 1)
)

knitr::kable(results,
             caption = paste("Benchmark results: processing time and",
                             "peak RAM (peakRAM)"))
```

Table 3: Benchmark results: processing time and peak RAM (peakRAM)

Scenario	Backend	Processing	Access	Time_s	Peak_Mem_Mb
(a)	ChromBackendMemory	Immediate	All	0.337	4.6
(b)	ChromBackendMemory	Immediate	Single	0.372	4.4
(c)	ChromBackendMemory	Lazy	All	0.119	1.1
(d)	ChromBackendMemory	Lazy	Single	0.013	0.9

Scenario	Backend	Processing	Access	Time_s	Peak_Mem_Mb
(e)	ChromBackendSpectra	Lazy	All	11.377	279.9
(f)	ChromBackendSpectra	Lazy	Single	0.975	210.3

```r
# Backend mapping for color encoding (only 2 colors, matching the design)
results$backend_short <- ifelse(results$Backend == "ChromBackendMemory",
                                "Memory", "Disk")

# Build descriptor strings: "(a)  Memory · Immediate · All"
results$row_label <- sprintf("(%s)  %s · %s · %s",
                             gsub("[()]", "", results$Scenario),
                             results$backend_short,
                             results$Processing,
                             results$Access)

# Reading order: a at top, f at bottom -> reverse for ggplot's bottom-up axis
row_order <- rev(results$row_label)

# Pre-compute display labels for each metric
# Time: ms if <1s, otherwise s. Memory: Mb.
results$time_label <- ifelse(results$Time_s < 1,
                             paste0(round(results$Time_s * 1000), " ms"),
                             paste0(formatC(results$Time_s, format = "f",
                                            digits = 1), " s"))
results$mem_label <- paste0(formatC(results$Peak_Mem_Mb, format = "f",
                                    digits = 1), " MB")

# Threshold for "below axis scale" treatment in the time panel
TIME_BELOW <- 0.5
results$time_below_threshold <- results$Time_s < TIME_BELOW

# Reshape with metric-specific helper columns
plot_df <- results |>
  pivot_longer(cols = c(Time_s, Peak_Mem_Mb),
               names_to = "Metric", values_to = "Value") |>
  mutate(
    Metric = factor(case_match(Metric,
                               "Peak_Mem_Mb" ~ "Peak RAM (MB)",
                               "Time_s" ~ "Execution time (s)"),
                    levels = c("Peak RAM (MB)",
                               "Execution time (s)")),
    display_label = ifelse(Metric == "Execution time (s)",
                           time_label, mem_label),
    # Visible-minimum bar for sub-threshold time values:
    # use a small fraction of each panel's max so the bar is visible
    is_below = Metric == "Execution time (s)" & time_below_threshold,
```

```r
    plot_value = ifelse(is_below,
                        max(Value[Metric == "Execution time (s)"]) * 0.015,
                        Value),
    label_color = ifelse(is_below, "red", "black"),
    row_label = factor(row_label, levels = row_order)
  )

# Backend color palette (matches the SVG figure)
backend_colors <- c(Memory = "#2E86AB", Disk = "#E07B39")

ggplot(plot_df, aes(x = plot_value, y = row_label, fill = backend_short)) +
  geom_col(width = 0.62) +
  geom_text(aes(label = display_label, colour = label_color),
            hjust = -0.08, size = 3.3, fontface = "bold",
            show.legend = FALSE) +
  scale_colour_identity() +
  scale_fill_manual(values = backend_colors,
                    breaks = c("Memory", "Disk"),
                    labels = c("Memory backend (ChromBackendMemory)",
                               "Disk backend (ChromBackendSpectra)")) +
  scale_x_continuous(expand = expansion(mult = c(0, 0.22))) +
  facet_wrap(~ Metric, scales = "free_x") +
  labs(x = NULL, y = NULL, fill = "Backend") +
  theme_minimal(base_size = 12) +
  theme(
    panel.grid.major.y = element_blank(),
    panel.grid.minor   = element_blank(),
    panel.grid.major.x = element_line(colour = "grey92"),
    strip.text         = element_text(face = "bold", size = 12),
    legend.position    = "bottom",
    legend.title       = element_text(face = "bold"),
    axis.text.y        = element_text(family = "mono", hjust = 0)
  )
```

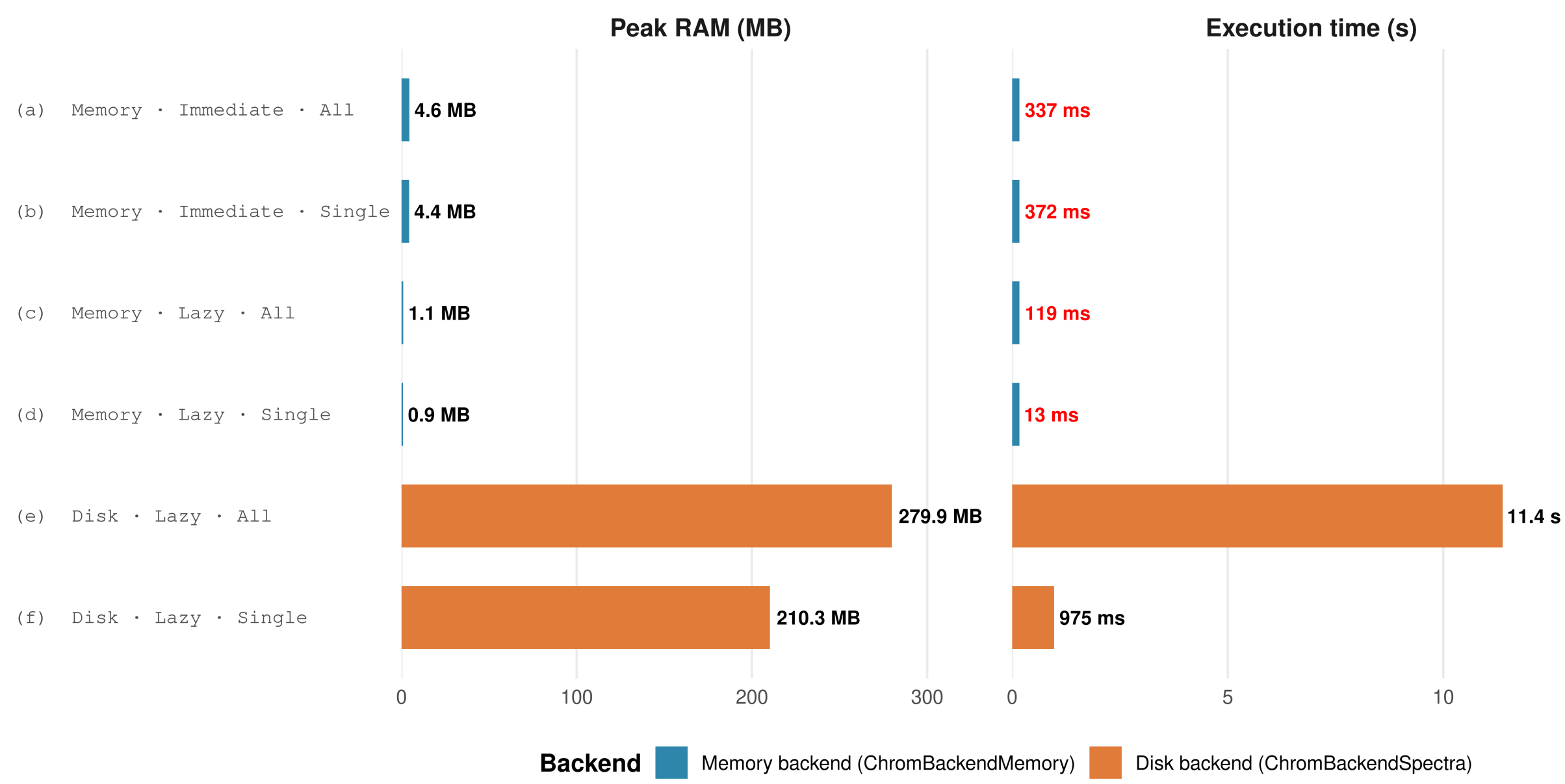

Figure 1: Execution time and peak RAM (peakRAM) for a three-step chromatographic processing pipeline (intensity filtering, linear imputation, retention-time windowing) under six scenarios combining backend, execution strategy, and access pattern. Scenario labels (a)-(f) match Table 1. Memory backend (ChromBackendMemory) is shown in blue, on-disk backend (ChromBackend-Spectra) in orange. Times below 0.5 s are drawn at a visible minimum width and labelled in red to flag values below the axis resolution.

Conclusion

The benchmark shows how execution strategy, backend choice, and access pattern interact to determine both execution time and peak memory.

Immediate vs. lazy evaluation

Comparing immediate (a, b) with lazy in-memory (c, d) isolates the effect of the processing queue alone. Immediate processing forces three full passes over the entire dataset, taking 0 s. Lazy evaluation with full access (c 0 s) collapses those three passes into one, yielding a 3× speed-up at comparable peak memory (1 vs 5 MB).

Lazy evaluation combined with subset access (d 0.01 s) goes much further: because the `[` operator carries the pending queue without evaluating it, only one chromatogram is processed. The resulting speed-up against the immediate baseline (a) is **26×** - i.e. 1.4 orders of magnitude.

The cost of on-disk full access

Scenario (e) - on-disk, lazy, full access - is the most memory-intensive of the six (peak RAM 280 MB), roughly 60.8× the peak footprint of the in-memory equivalents, and slower than lazy in-memory full access (e 11 s vs c 0 s). *ChromBackendSpectra* has to read raw spectra from all 10 mzML files and build chromatographic peaks at access time, which requires substantial temporary memory; *ChromBackendMemory* holds pre-summarised, compact chromatograms that are already in RAM.

Subset access on disk (f 1 s, peak 210 MB) drastically reduces both time and memory compared to (e), because only one file's spectra are read. It remains slower and more memory-hungry than the equivalent in-memory subset (d 0.01 s, peak 1 MB).

Practical workflow recommendations

The results suggest a two-stage workflow for large datasets:

1. **Start on disk when the full dataset does not fit in memory.** *ChromBackendSpectra* avoids loading all raw data upfront. Combined with lazy evaluation, it lets the user build a processing pipeline and explore targeted subsets - individual chromatograms, RT windows, specific samples - without paying the cost of full materialisation.

2. **Switch the subset of interest to the in-memory backend.** Once the relevant chromatograms have been identified, calling *setBackend()* on the subset converts it to *ChromBackendMemory*. Subsequent operations - repeated access, visualisation, iterative analysis - then benefit from in-memory speed without I/O overhead.

Crucially, **repeated full-data access from an on-disk backend should be avoided**. Scenario (e) shows it to be both the slowest and the most memory-intensive pattern. The on-disk backend is designed for selective, lazy access - not as a substitute for in-memory storage when the full dataset is needed repeatedly.

In summary, the lazy processing queue is not merely a convenience - it is a performance mechanism. By deferring computation until data are accessed, subsetting filters *what gets processed and loaded*, not just what gets returned. Combined with backend switching, this enables interactive exploration of datasets that exceed available memory while still achieving almost immediate response times for targeted queries.

References

1. Rainer J, Gatto L, Gibb S. A Modular and Expandable Ecosystem for Metabolomics Data Annotation in R. *Metabolites*. 2022;12(2):173. doi:10.3390/metabo12020173

2. Gatto L, Gibb S, Rainer J. MSnbase, Efficient and Elegant R-Based Processing and Visualization of Raw Mass Spectrometry Data. *J Proteome Res.* 2020; 19(4):1063–1069. doi:10.1021/acs.jproteome.0c00313

Key results for manuscript

Table 4: Headline numbers used in the main manuscript. Re-check these against the paper after each rendering.

Scenario	Metric	Value
(a) Memory + Immediate + All	elapsed (s)	0.34
(a) Memory + Immediate + All	peak memory (MB)	4.6
(b) Memory + Immediate + Single	elapsed (s)	0.37
(b) Memory + Immediate + Single	peak memory (MB)	4.4

Scenario	Metric	Value
(c) Memory + Lazy + All	elapsed (s)	0.12
(c) Memory + Lazy + All	peak memory (MB)	1.1
(d) Memory + Lazy + Single	elapsed (s)	0.01
(d) Memory + Lazy + Single	peak memory (MB)	0.9
(e) Disk + Lazy + All	elapsed (s)	11.38
(e) Disk + Lazy + All	peak memory (MB)	279.9
(f) Disk + Lazy + Single	elapsed (s)	0.98
(f) Disk + Lazy + Single	peak memory (MB)	210.3
Derived	Lazy speed-up: (a) elapsed / (d) elapsed	26x (10^1.4)
Derived	Lazy speed-up: (a) elapsed / (c) elapsed	2.8x
Derived	Memory reduction: (e) peak / (d) peak	311.0x

Session Info

```
sessionInfo()
```

```
R version 4.6.0 (2026-04-24)
Platform: x86_64-pc-linux-gnu
Running under: Arch Linux

Matrix products: default
BLAS:   /home/jo/R/local/R-4.6.0/lib64/R/lib/libRblas.so
LAPACK: /usr/lib/liblapack.so.3.12.0  LAPACK version 3.12.0

locale:
 [1] LC_CTYPE=en_US.UTF-8       LC_NUMERIC=C
 [3] LC_TIME=en_US.UTF-8        LC_COLLATE=en_US.UTF-8
 [5] LC_MONETARY=en_US.UTF-8    LC_MESSAGES=en_US.UTF-8
 [7] LC_PAPER=en_US.UTF-8       LC_NAME=C
 [9] LC_ADDRESS=C               LC_TELEPHONE=C
[11] LC_MEASUREMENT=en_US.UTF-8 LC_IDENTIFICATION=C

time zone: Europe/Rome
tzcode source: system (glibc)

attached base packages:
[1] stats4    stats     graphics  grDevices utils     datasets  methods
[8] base

other attached packages:
 [1] ggplot2_4.0.3              tidyr_1.3.2
 [3] dplyr_1.2.1                peakRAM_1.0.2
 [5] MsBackendMetaboLights_1.6.1 Spectra_1.22.2
 [7] S4Vectors_0.50.1           BiocGenerics_0.58.1
 [9] generics_0.1.4             Chromatograms_1.3.3
[11] ProtGenerics_1.44.0        BiocParallel_1.46.0
```

```
loaded via a namespace (and not attached):
 [1] gtable_0.3.6           xfun_0.60              httr2_1.3.0
 [4] Biobase_2.72.0         vctrs_0.7.3            tools_4.6.0
 [7] curl_7.1.0             parallel_4.6.0         tibble_3.3.1
[10] RSQLite_3.53.3         cluster_2.1.8.3        blob_1.3.0
[13] pkgconfig_2.0.3        data.table_1.18.4      dbplyr_2.6.0
[16] RColorBrewer_1.1-3     S7_0.2.2               lifecycle_1.0.5
[19] compiler_4.6.0         farver_2.1.2           progress_1.2.3
[22] codetools_0.2-20       ncdf4_1.24             clue_0.3-68
[25] htmltools_0.5.9        yaml_2.3.12            pillar_1.11.1
[28] crayon_1.5.3           MASS_7.3-66            cachem_1.1.0
[31] MetaboCoreUtils_1.20.1 tidyselect_1.2.1       digest_0.6.39
[34] purrr_1.2.2            labeling_0.4.3         fastmap_1.2.0
[37] grid_4.6.0             cli_3.6.6              magrittr_2.0.5
[40] dichromat_2.0-1        withr_3.0.3            prettyunits_1.2.0
[43] filelock_1.0.3         scales_1.4.0           bit64_4.8.2
[46] rmarkdown_2.31         bit_4.6.0              otel_0.2.0
[49] hms_1.1.4              memoise_2.0.1          evaluate_1.0.5
[52] knitr_1.51             IRanges_2.46.0         BiocFileCache_3.2.0
[55] rlang_1.3.0            Rcpp_1.1.2             glue_1.8.1
[58] DBI_1.3.0              mzR_2.46.0             jsonlite_2.0.0
[61] R6_2.6.1               fs_2.1.0               MsCoreUtils_1.24.0
```
