# Supplementary material for: Chromatograms: A Modular Infrastructure for Scalable Chromatographic Data Handling in R
Source: Anal Chem. 2026 Sep 17;98(38):28337–44. doi: 10.1021/acs.analchem.6c03926 (PMC13629298; doi:10.1021/acs.analchem.6c03926)
Supplement: Supplementary file 3 [file ac6c03926_si_003.pdf]

Interoperable, cohort-scale targeted reanalysis with Chromatograms

Supporting Information for: *Chromatograms: A modular infrastructure for scalable chromatographic data handling in R*

Philippine Louail[1,2,*], Laurent Gatto[3], Sebastian Gibb[4], Johannes Rainer[1]

[1] Institute for Biomedicine, Eurac Research, Bolzano, Italy [2] Chair for Bioinformatics, Friedrich-Schiller-University Jena, 07743 Jena, Germany [3] Computational Biology and Bioinformatics, de Duve Institute, UCLouvain, Brussels, Belgium [4] Department of Anaesthesiology and Intensive Care, University Medicine Greifswald, Greifswald, Germany

* Corresponding author: philippine.louail@outlook.com

Table of contents

Motivation . . . S1
System Information . . . S2
1. Target compounds . . . S2
2. Import and subset . . . S3
    2.1 Locating an unannotated retention time (SM 28:1) . . . S3
3. Extract EICs with *chromExtract()* . . . S5
4. Quantify and quality-assess the EICs . . . S6
5. EIC overlay . . . S7
    5.1 Retention-time deviation across the cohort . . . S8
6. Scaling across the cohort . . . S9
Key takeaways . . . S11
Session Info . . . S11

Motivation

Once a study has annotated a set of compounds (m/z and retention time), those annotations can be revisited in the raw data: pulled out and re-quantified, across the whole cohort or in new samples. Doing this the untargeted way - re-running peak detection, alignment and correspondence - is expensive. The Metabonaut large-scale analysis tutorial performs exactly that untargeted *xcms* workflow on the cohort used here, and it takes many hours of processing and hundreds of GB of intermediate data.

`Chromatograms::chromExtract()` offers a lightweight, targeted alternative. Given a plain peak table of m/z / retention-time windows, it pulls the corresponding extracted-ion chromatograms (EICs) directly from the raw data - no peak detection, no alignment - and returns a `Chromatograms` object. The recovered EICs are then quantified and quality-assessed with the package's own functions (`peakBoundary()`) together with *MsQuality*, and visualised with `plotChromatogramsOverlay()`. Because the workflow consumes *published annotations* and *raw public data*, it is tool-agnostic: the peak table can come from any study or software (*MZmine*, *MS-DIAL*, *xcms*, a vendor export, or, as here, a published table), and the resulting `Chromatograms` object flows on into any downstream analysis.

This document reanalyses **MTBLS93** (Ganna *et al.* 2014, *PLOS Genetics*, doi:10.1371/journal.pgen.1004801; the TwinGene cohort, ~4063 MS1 samples), recovering the coronary-heart-disease lipid biomarkers the study annotated. It demonstrates, using native `Chromatograms` functionality throughout:

1. targeted EIC extraction of the published biomarkers with `chromExtract()`;
2. quantification and quality assessment with `peakBoundary()` and *MsQuality*;
3. locating an annotation with no published retention time (SM 28:1) directly from the data;
4. how targeted extraction scales across the cohort (number of files).

```
library(Chromatograms)
library(Spectra)
library(MsBackendMetaboLights)
library(MsQuality)
library(BiocParallel)
library(peakRAM)

## Run serially so scaling reflects design, not the number of workers.
register(SerialParam())
```

```
RT_RANGE <- c(20, 900)   # elution window (s)
```

System Information

```
knitr::kable(
  data.frame(
    Item  = c("CPU Cores", "R Version", "Platform", "Chromatograms Version"),
    Value = c(
      parallel::detectCores(),
      R.version$version.string,
      R.version$platform,
      as.character(packageVersion("Chromatograms"))
    )
  ),
  caption = "System information"
)
```

Table 1: System information

Item	Value
CPU Cores	24
R Version	R version 4.6.0 (2026-04-24)
Platform	x86_64-pc-linux-gnu
Chromatograms Version	1.3.3

1. Target compounds

The four replicated biomarkers from the study (MSI level 1). Both the m/z (`[M+H]+`) and the retention times are taken from the article's targeted MS/MS confirmation figure (S2 Figure), which

reports elution times in minutes: LysoPC 18:1 at 6.37 min (382.2 s), LysoPC 18:2 at 5.78 min (346.8 s) and MG 18:2 at 6.40 min (384.0 s). SM 28:1 is confirmed by accurate mass (m/z 661.526) but the article gives **no** retention time for it; Section 2.1 shows how to locate it directly from the data before it joins the other targets.

```
targets <- data.frame(
  name = c("LysoPC_18:1", "LysoPC_18:2", "MG_18:2", "SM_28:1"),
  mz   = c(522.356, 520.341, 337.269, 661.526),
  rt   = c(382.2, 346.8, 384.0, NA),   # S2 Figure (min x 60), SM 28:1 not given
  stringsAsFactors = FALSE
)
targets$mzmin <- targets$mz - 0.01
targets$mzmax <- targets$mz + 0.01
targets$rtmin <- targets$rt - 20
targets$rtmax <- targets$rt + 20

knitr::kable(targets, digits = 3, caption = "Annotated target biomarkers")
```

Table 2: Annotated target biomarkers

name	mz	rt	mzmin	mzmax	rtmin	rtmax
LysoPC__18:1	522.356	382.2	522.346	522.366	362.2	402.2
LysoPC__18:2	520.341	346.8	520.331	520.351	326.8	366.8
MG__18:2	337.269	384.0	337.259	337.279	364.0	404.0
SM__28:1	661.526	NA	661.516	661.536	NA	NA

2. Import and subset

MTBLS93 holds ~4063 MS1 files and ~800 GB of raw data. This data is retrieved below and lazy-loaded as a `Spectra` object (i.e., without loading the actual MS data into memory). Retention time is restricted to the elution window to cut I/O.

```
sps <- Spectra(source = MsBackendMetaboLights(), mtblsId = "MTBLS93",
               filePattern = "01.CDF$")
sps <- filterRt(sps, rt = RT_RANGE)
files <- unique(dataOrigin(sps))
```

Table 3: MTBLS93 MS1 data set

Item	Value
Files	4063
Total spectra	8056661
RT range (s)	20.3 - 899.8

2.1 Locating an unannotated retention time (SM 28:1)

The article confirms SM 28:1 by accurate mass and MS/MS but does not report a retention time for it. Rather than borrow a value from an untargeted result, we locate it directly with the same

targeted machinery, used here as a discovery step: extract the EIC across the *entire* elution window at the target m/z from a data subset (n = 10 files), see where the peak falls, then narrow the retention-time window and re-extract.

```
sm <- which(targets$name == "SM_28:1")

sps_sub <- filterDataOrigin(sps, files[1:10])

## SM 28:1 over the full elution window, one row per file
sm_table <- data.frame(
  name = "SM_28:1", mzMin = targets$mzmin[sm], mzMax = targets$mzmax[sm],
  rtMin = RT_RANGE[1], rtMax = RT_RANGE[2], msLevel = 1L,
  dataOrigin = files[1:10])
sm_chr <- filterEmptyChromatograms(
    chromExtract(Chromatograms(sps_sub, summarize.method = "max"),
                 peak.table = sm_table,
                 by = c("msLevel", "dataOrigin"))) |>
    setBackend(backend = ChromBackendMemory())

## consensus apex across the cohort: per-file apex retention time, median
apex_rt <- mapply(function(int, rt) rt[which.max(int)],
                  intensity(sm_chr), rtime(sm_chr))
sm_rt <- round(median(apex_rt), 2)
```

Over the full window a single peak stands out; its consensus apex across the files is at 579.42 s. We record that retention time and narrow the EIC we already extracted - `filterPeaksData()` trims each trace to the located window, no re-extraction from the raw data needed.

```
targets$rt[sm]    <- sm_rt
targets$rtmin[sm] <- sm_rt - 20
targets$rtmax[sm] <- sm_rt + 20

sm_chr2 <- filterPeaksData(sm_chr, variables = "rtime",
                           ranges = c(sm_rt - 20, sm_rt + 20))

par(mfrow = c(1, 2), mar = c(4, 4, 3, 1))
Chromatograms::plotChromatogramsOverlay(
  sm_chr, col = "#0000AA40", main = "SM 28:1 - full window")
abline(v = sm_rt, col = "red", lty = 2)
Chromatograms::plotChromatogramsOverlay(
  sm_chr2, col = "#0000AA40",
  main = sprintf("SM 28:1 - narrowed (rt = %.1f s)", sm_rt))
abline(v = sm_rt, col = "red", lty = 2)
```

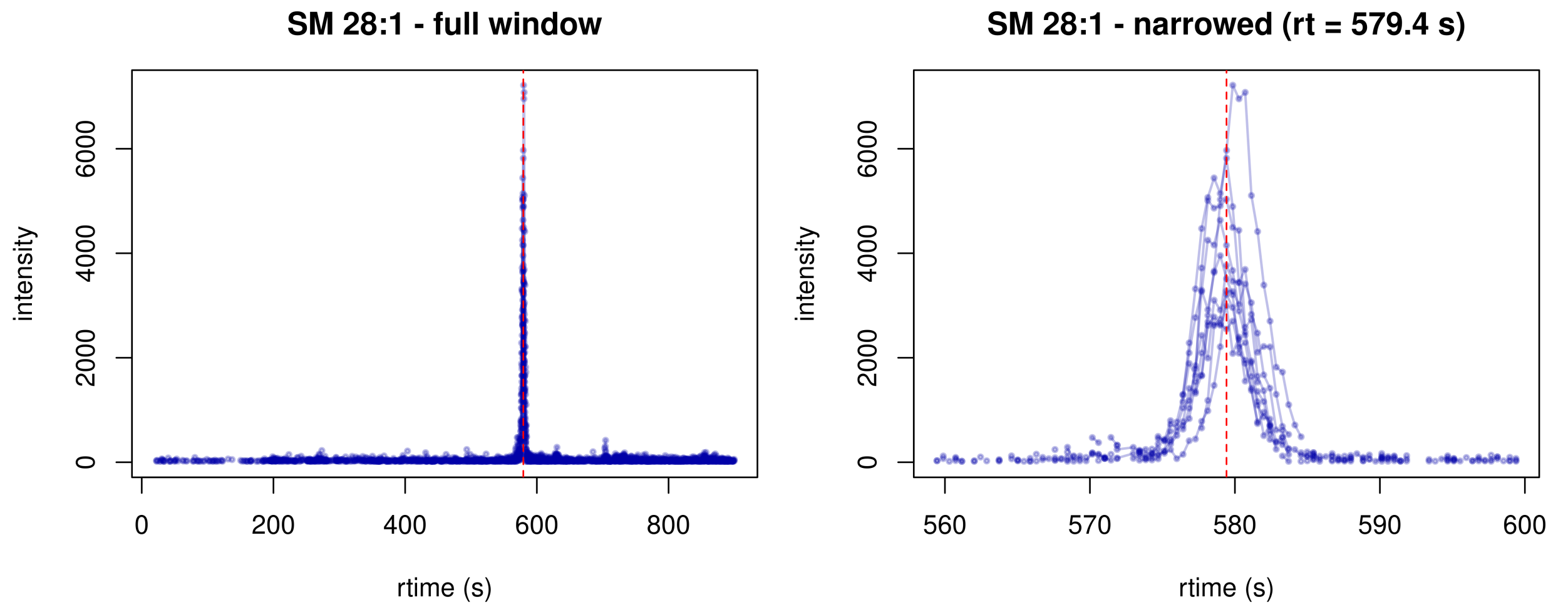

Figure 1: SM 28:1 EIC over the full elution window (left) and narrowed to the located peak (right). Dashed line: consensus apex.

With its retention time fixed at 579.42 s, SM 28:1 joins the other three targets and is treated identically from here on.

3. Extract EICs with *chromExtract()*

The target table is expanded to one row per (target, file). `chromExtract()` requires `mzMin`, `mzMax`, `rtMin`, `rtMax` and the `by` columns. Every other column (here the compound name and expected m/z / rt) is carried through and preserved on the output `chromData`.

```
peak_table <- data.frame(
  name = targets$name, target_mz = targets$mz, target_rt = targets$rt,
  mzMin = targets$mzmin, mzMax = targets$mzmax,
  rtMin = targets$rtmin, rtMax = targets$rtmax,
  msLevel = 1L, dataOrigin = rep(files, each = nrow(targets)))
```

```
chr <- chromExtract(Chromatograms(sps, summarize.method = "max"),
                    peak.table = peak_table,
                    by = c("msLevel", "dataOrigin"))
chr <- setBackend(chr, backend = ChromBackendMemory())
chr <- filterEmptyChromatograms(chr)
chr
```

```
Chromatographic data (Chromatograms) with 16252 chromatograms in a ChromBackendMemory backend:
  chromIndex msLevel  mz
1         NA       1 Inf
2         NA       1 Inf
3         NA       1 Inf
4         NA       1 Inf
5         NA       1 Inf
6         NA       1 Inf
... 15 more  chromatogram variables/columns
```

```
... 2 peaksData variables
Processing:
 Switch backend from ChromBackendSpectra to ChromBackendMemory [Wed Aug  5 09:59:00 2026]
 Filter: removed empty chromatograms. [Wed Aug  5 09:59:00 2026]
```

The external metadata are preserved on `chromData`:

Table 4: chromData of extracted EICs (first 6 rows)

name	target_mz	target_rt	mzMin	mzMax	dataOrigin
LysoPC_18:1	522.356	382.20	522.346	522.366	021228_TwinGene_000101.CDF
LysoPC_18:2	520.341	346.80	520.331	520.351	021228_TwinGene_000101.CDF
MG_18:2	337.269	384.00	337.259	337.279	021228_TwinGene_000101.CDF
SM_28:1	661.526	579.42	661.516	661.536	021228_TwinGene_000101.CDF
LysoPC_18:1	522.356	382.20	522.346	522.366	021228_TwinGene_000201.CDF
LysoPC_18:2	520.341	346.80	520.331	520.351	021228_TwinGene_000201.CDF

4. Quantify and quality-assess the EICs

Rather than integrate the traces by hand, we use the package's own peak functions. `peakBoundary()` locates the retention-time boundaries of the tallest peak in each EIC, and *MsQuality*'s `calculateMetrics()` computes abundance and shape metrics (apex intensity, area under the trace, peak width at half maximum). Both are attached to `chromData`.

```r
bounds <- peakBoundary(chr)

metrics <- calculateMetrics(
    chr,
    metrics = c("maxIntensity", "areaUnderTic", "xicFwhm"),
    na.rm = TRUE, filterEmptyObject = FALSE, baselineQuantile = 0.1
)
metrics <- as.data.frame(metrics, row.names = NULL)

chromData(chr) <- cbind(chromData(chr),
                        left_boundary = bounds[, "left_boundary"],
                        right_boundary = bounds[, "right_boundary"],
                        metrics)
```

The result is a single `data.frame` in which the carried-through annotation (`name`, `target_rt`), the peak boundaries and the quality metrics sit side by side - one row per extracted EIC.

Table 5: chromData: annotation, peak boundaries and metrics (rows 5-10)

name	target_rt	left_boundary	right_boundary	maxIntensity	areaUnderTic	xicFwhm
LysoPC_18:1	382.20	375.89	387.46	465345.50	6429341.65	4.07
LysoPC_18:2	346.80	341.10	352.50	580488.50	6361619.15	3.73
MG_18:2	384.00	380.18	388.32	1654.00	19337.23	3.35
SM_28:1	579.42	576.00	584.14	7215.62	66885.12	3.36

name	target_rt	left_boundary	right_boundary	maxIntensity	areaUnderTic	xicFwhm
LysoPC_18:1	382.20	374.61	387.46	462720.00	6000868.97	4.03
LysoPC_18:2	346.80	340.67	352.93	607204.50	6655301.38	3.72

Per-target summary. The LysoPCs are high-abundance and recovered in every sample, MG 18:2 is weaker. The peak boundaries from `peakBoundary()` bracket the annotated retention time in every case.

Table 6: Recovery, quantification and quality per target

name	n_files	with_signal	median_apex	median_fwhm_s	target_rt
LysoPC_18:1	4063	4063	509825	4.06	382.20
LysoPC_18:2	4063	4063	793327	3.70	346.80
MG_18:2	4063	4063	1731	3.12	384.00
SM_28:1	4063	4063	3857	3.47	579.42

5. EIC overlay

`plotChromatogramsOverlay()` overlays the extracted EICs across the subset for each target. A clear peak appears near the annotated retention time (dashed line), with the sample-to-sample retention-time drift characteristic of this cohort.

```r
cd <- chromData(chr)
par(mfrow = c(2, ceiling(nrow(targets) / 2)), mar = c(4, 4, 3, 1))
for (nm in targets$name) {
    tgt <- targets[targets$name == nm, ]
    idx <- which(cd$name == nm)
    Chromatograms::plotChromatogramsOverlay(
                       chr[idx], col = "#0000AA40",
                       main = sprintf("%s (m/z %.3f)", nm, tgt$mz))
    abline(v = tgt$rt, col = "grey60", lty = 2)
}
```

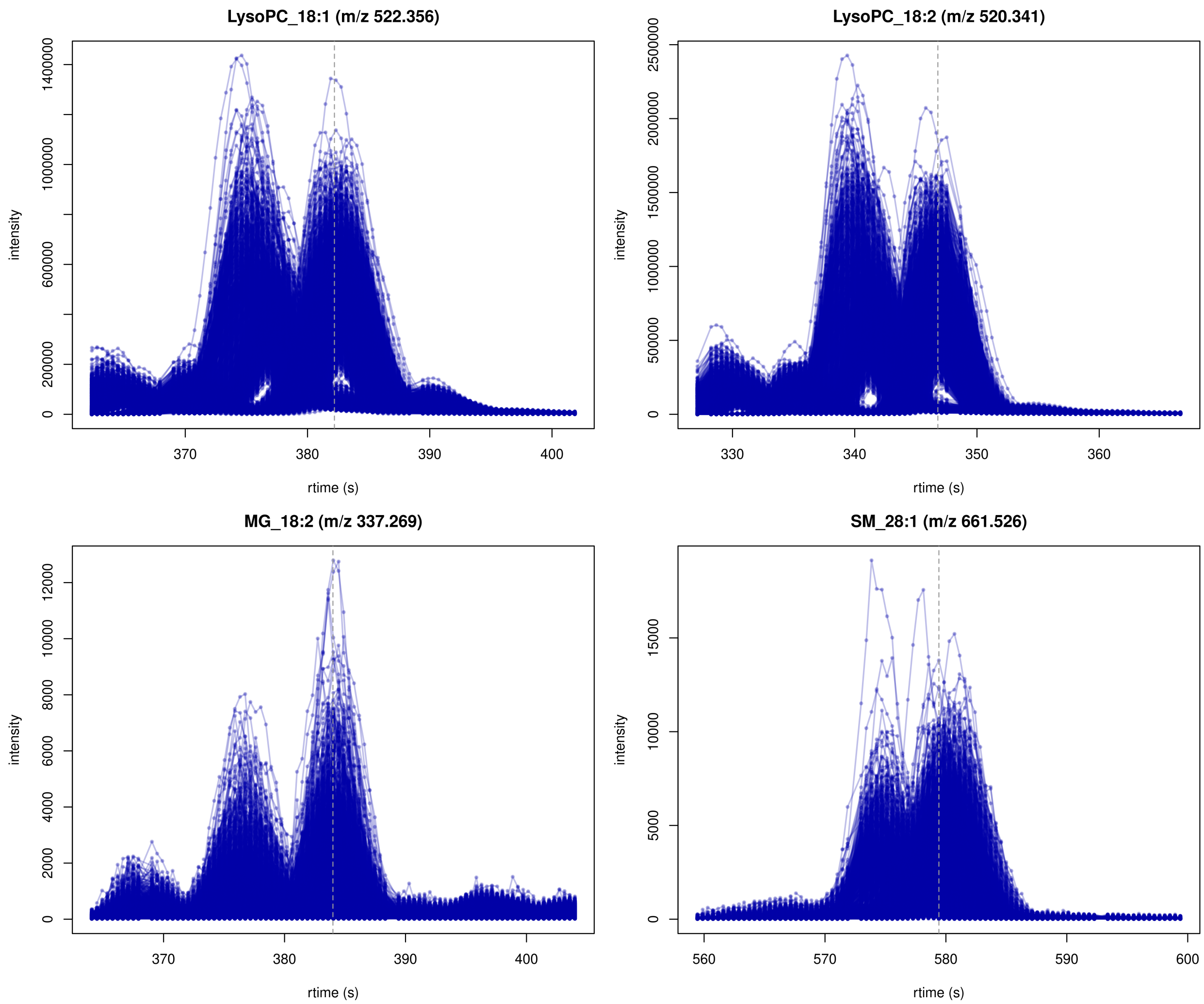

Figure 2: Extracted-ion chromatograms of the annotated biomarkers across the subset (`plotChromatogramsOverlay()`). Dashed line: annotated retention time.

Plotting the EICs for the full data set allows us also to identify possible batch effects. Indeed, the signal from the 4 compounds seems to be shifted along the retention time axis in a subset of samples.

5.1 Retention-time deviation across the cohort

That visual impression can be turned into a quick quality check: for a single biomarker, take the retention time of the apex (`rtime` at maximum intensity) in each sample and plot the distribution. The apex times split into two clusters, confirming a systematic retention-time deviation between subsets of the cohort (a batch effect) rather than random scatter.

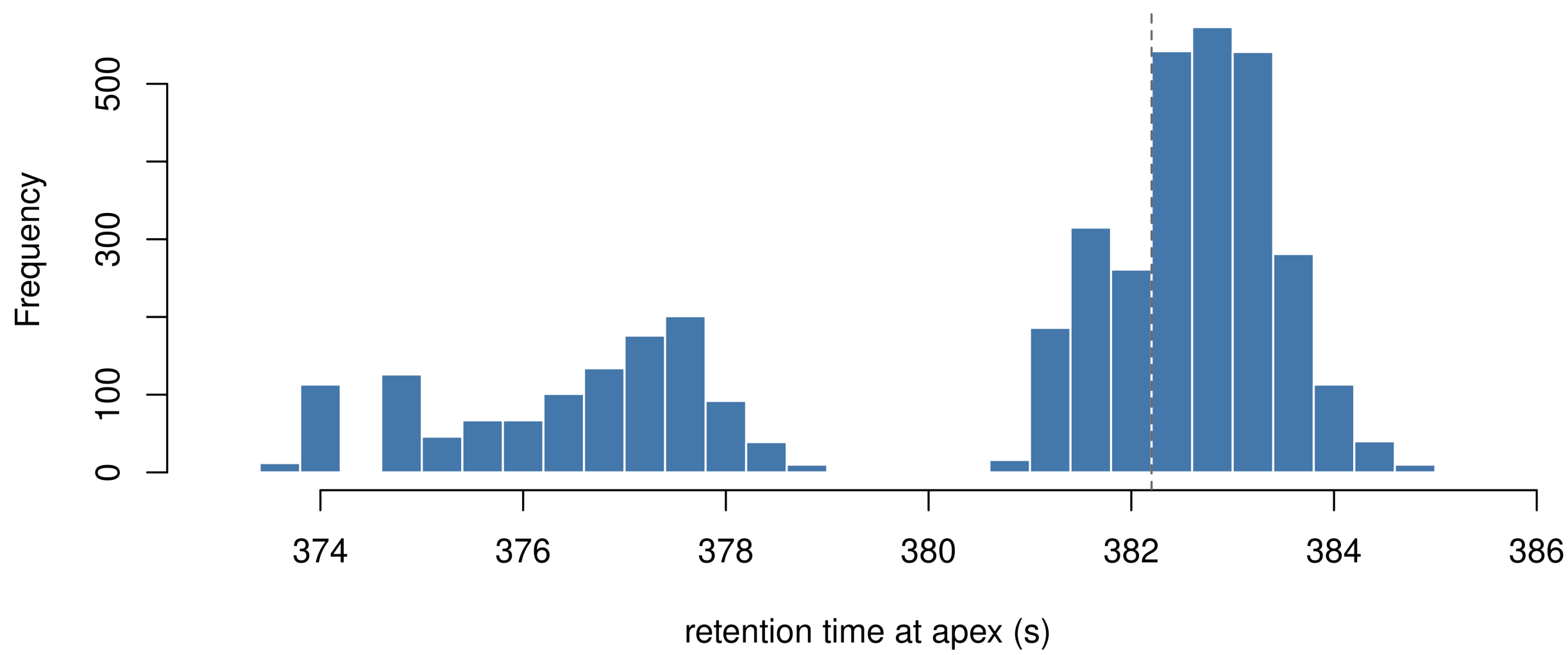

Figure 3: Distribution of the per-sample apex retention time for LysoPC 18:1. The two well-separated modes are the batch retention-time deviation across the cohort. Dashed line: annotated retention time.

6. Scaling across the cohort

The targeted workflow processes each file independently, so it scales with the number of files. We sweep the file count and record the time and memory usage to extract all target EICs (materialised into memory with `setBackend()`), using `peakRAM`. The `extract_+_metrics_s` column adds the time to run `peakBoundary()` and `calculateMetrics()` on top of the extraction, i.e. the cost of the full quantified workflow. Both are measured over the same four biomarkers.

```
file_counts <- c(10, 50, 100, 500, 1000, 2000, 4000)

throughput <- do.call(rbind, lapply(file_counts, function(n) {
  fsub <- files[seq_len(n)]
  ssub <- sps[dataOrigin(sps) %in% fsub]
  pt <- peak_table[peak_table$dataOrigin %in% fsub, ]
  pr <- peakRAM(cn <- setBackend(Chromatograms(ssub, chromData = pt,
                                               summarize.method = "max"),
                                 ChromBackendMemory()))
  cn <- filterEmptyChromatograms(cn)
  metrics_s <- system.time({
    peakBoundary(cn)
    calculateMetrics(cn, metrics = c("maxIntensity", "areaUnderTic", "xicFwhm"),
                     na.rm = TRUE, filterEmptyObject = FALSE,
                     baselineQuantile = 0.1)
  })[["elapsed"]]
  data.frame(n_files = n,
             extract_s = pr$Elapsed_Time_sec,
             `extract_+_metrics_s` = pr$Elapsed_Time_sec + metrics_s,
             peak_MB = pr$Peak_RAM_Used_MiB,
             total_MB = pr$Total_RAM_Used_MiB, check.names = FALSE)
```

```
}))
knitr::kable(throughput, digits = 2, row.names = FALSE,
             caption = "chromExtract throughput vs number of files")
```

Table 7: chromExtract throughput vs number of files

n_files	extract_s	extract_+_metrics_s	peak_MB	total_MB
10	7.18	7.21	4498.6	0.0
50	36.66	36.76	4480.9	0.3
100	72.75	72.95	4458.6	0.6
500	377.71	378.70	4279.9	3.0
1000	767.97	769.92	4056.7	5.9
2000	1526.15	1530.99	3610.3	11.9
4000	2928.90	2937.09	5570.1	24.0

```
par(mfrow = c(1, 2))
plot(throughput$n_files, throughput$extract_s, type = "b", pch = 19,
     col = "#1B9E77", xlab = "number of files", ylab = "seconds",
     main = "Extraction time")
grid()
plot(throughput$n_files, throughput$total_MB, type = "b", pch = 19,
     col = "#D95F02", xlab = "number of files", ylab = "object size (MiB)",
     main = "Total memory")
grid()
```

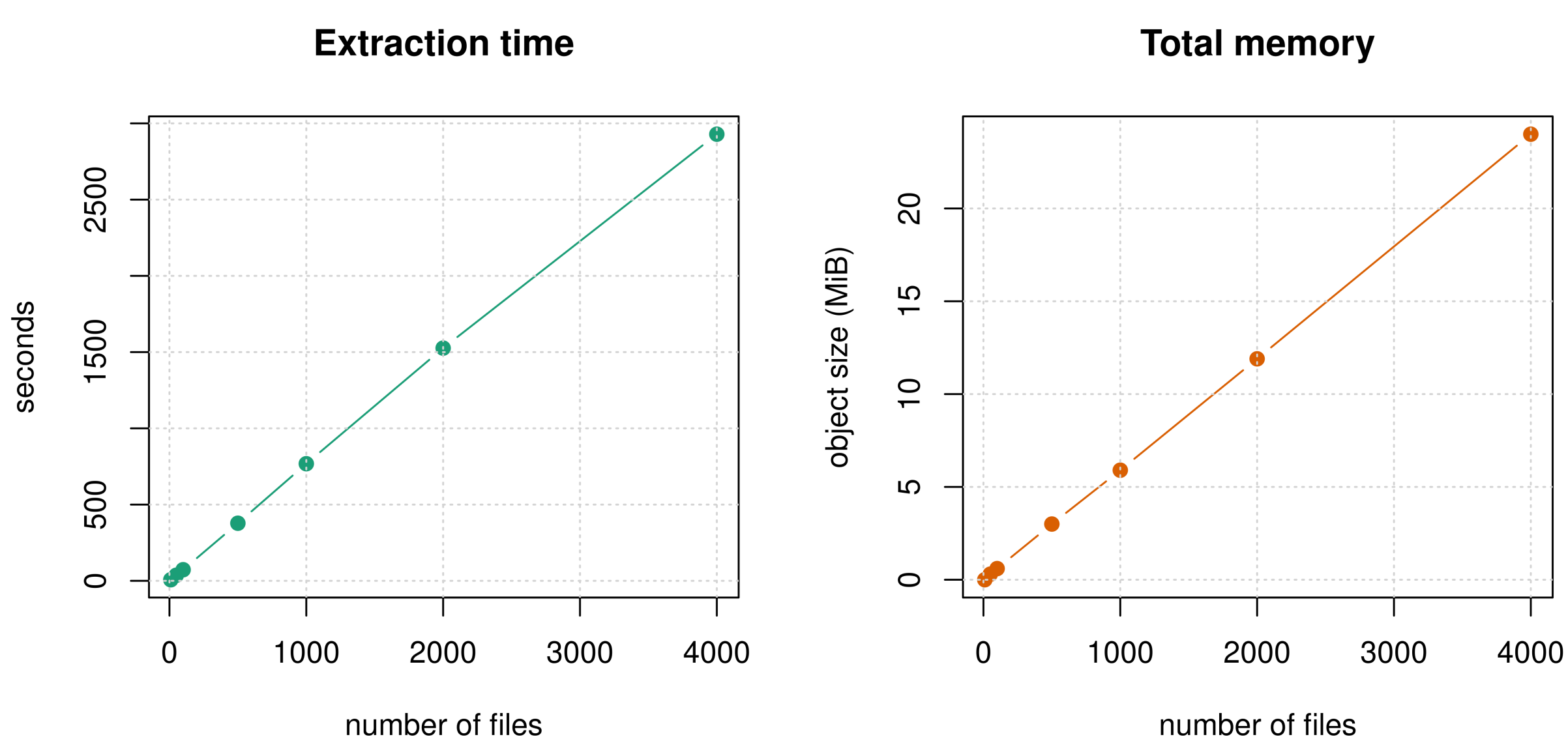

Figure 4: Targeted extraction time and object's size in memory vs number of files.

Key takeaways

Starting from a study's published annotations (m/z + retention time) and its raw public files, `chromExtract()` recovers the target compounds in a memory efficient way across a cohort's full data in a single pass.

The untargeted route on the same cohort (the Metabonaut large-scale-analysis tutorial) requires many hours of *xcms* preprocessing. Targeted reanalysis with `chromExtract()` is a lightweight, annotation-driven alternative.

Session Info

```r
sessionInfo()
```

```
R version 4.6.0 (2026-04-24)
Platform: x86_64-pc-linux-gnu
Running under: Arch Linux

Matrix products: default
BLAS:   /home/jo/R/local/R-4.6.0/lib64/R/lib/libRblas.so
LAPACK: /usr/lib/liblapack.so.3.12.0  LAPACK version 3.12.0

locale:
 [1] LC_CTYPE=en_US.UTF-8       LC_NUMERIC=C
 [3] LC_TIME=en_US.UTF-8        LC_COLLATE=en_US.UTF-8
 [5] LC_MONETARY=en_US.UTF-8    LC_MESSAGES=en_US.UTF-8
 [7] LC_PAPER=en_US.UTF-8       LC_NAME=C
 [9] LC_ADDRESS=C               LC_TELEPHONE=C
[11] LC_MEASUREMENT=en_US.UTF-8 LC_IDENTIFICATION=C

time zone: Europe/Rome
tzcode source: system (glibc)

attached base packages:
[1] stats4    stats     graphics  grDevices utils     datasets  methods
[8] base

other attached packages:
 [1] peakRAM_1.0.2               MsQuality_1.12.0
 [3] MsBackendMetaboLights_1.6.1 Spectra_1.22.2
 [5] S4Vectors_0.50.1            BiocGenerics_0.58.1
 [7] generics_0.1.4              Chromatograms_1.3.3
 [9] ProtGenerics_1.44.0         BiocParallel_1.46.0

loaded via a namespace (and not attached):
  [1] DBI_1.3.0                   httr2_1.3.0
  [3] testthat_3.3.2              rlang_1.3.0
  [5] magrittr_2.0.5              shinydashboard_0.7.3
  [7] clue_0.3-68                 otel_0.2.0
```

```
  [9] matrixStats_1.5.0           compiler_4.6.0
 [11] RSQLite_3.53.3              png_0.1-9
 [13] vctrs_0.7.3                 reshape2_1.4.5
 [15] stringr_1.6.0               pkgconfig_2.0.3
 [17] MetaboCoreUtils_1.20.1      crayon_1.5.3
 [19] fastmap_1.2.0               dbplyr_2.6.0
 [21] XVector_0.52.0              promises_1.5.0
 [23] rmarkdown_2.31              purrr_1.2.2
 [25] bit_4.6.0                   xfun_0.60
 [27] MultiAssayExperiment_1.38.0 MsDataHub_1.12.0
 [29] cachem_1.1.0                jsonlite_2.0.0
 [31] progress_1.2.3              blob_1.3.0
 [33] later_1.4.8                 DelayedArray_0.38.2
 [35] parallel_4.6.0              prettyunits_1.2.0
 [37] cluster_2.1.8.3             R6_2.6.1
 [39] stringi_1.8.7               RColorBrewer_1.1-3
 [41] brio_1.1.5                  GenomicRanges_1.64.0
 [43] Rcpp_1.1.2                  Seqinfo_1.2.0
 [45] SummarizedExperiment_1.42.0 knitr_1.51
 [47] IRanges_2.46.0              httpuv_1.6.17
 [49] Matrix_1.7-6                igraph_2.3.3
 [51] tidyselect_1.2.1            dichromat_2.0-1
 [53] abind_1.4-8                 yaml_2.3.12
 [55] codetools_0.2-20            curl_7.1.0
 [57] lattice_0.22-9              tibble_3.3.1
 [59] plyr_1.8.9                  withr_3.0.3
 [61] shiny_1.14.0                Biobase_2.72.0
 [63] KEGGREST_1.52.2             S7_0.2.2
 [65] evaluate_1.0.5              ontologyIndex_2.12
 [67] BiocFileCache_3.2.0         ExperimentHub_3.2.0
 [69] Biostrings_2.80.1           pillar_1.11.1
 [71] BiocManager_1.30.27         filelock_1.0.3
 [73] MatrixGenerics_1.24.0       ncdf4_1.24
 [75] plotly_4.12.1               BiocVersion_3.23.1
 [77] hms_1.1.4                   ggplot2_4.0.3
 [79] scales_1.4.0                R6P_0.4.0
 [81] MsExperiment_1.14.0         xtable_1.8-8
 [83] glue_1.8.1                  lazyeval_0.2.3
 [85] tools_4.6.0                 AnnotationHub_4.2.2
 [87] data.table_1.18.4           mzR_2.46.0
 [89] QFeatures_1.22.0            fs_2.1.0
 [91] grid_4.6.0                  jsonvalidate_1.5.0
 [93] tidyr_1.3.2                 MsCoreUtils_1.24.0
 [95] AnnotationDbi_1.74.0        cli_3.6.6
 [97] rappdirs_0.3.4              viridisLite_0.4.3
 [99] S4Arrays_1.12.0             dplyr_1.2.1
[101] AnnotationFilter_1.36.0     gtable_0.3.6
[103] digest_0.6.39               SparseArray_1.12.2
```

```
[105] htmlwidgets_1.6.4          farver_2.1.2
[107] memoise_2.0.1              htmltools_0.5.9
[109] lifecycle_1.0.5            httr_1.4.8
[111] mime_0.13                  rmzqc_0.7.0
[113] bit64_4.8.2                MASS_7.3-66
```
